# Supplementary material for: Unpacking musical beauty: Sound, emotion, and impact differences across expertise and personality
Source: PLoS One. 2025 Nov 14;20(11):e0335905. doi: 10.1371/journal.pone.0335905 (PMC12617921; doi:10.1371/journal.pone.0335905)
Supplement: S1 File — (DOCX) [file pone.0335905.s001.docx]

**S1 Supporting Information: The prevalence of codes**

The figures below show the rate of occurrence of each code relative to the number of text responses for each question: Feature (Fig A), Affect (Fig B), and Impact (Fig C).

**Fig A. The rate of occurrence of each code: Feature**

**
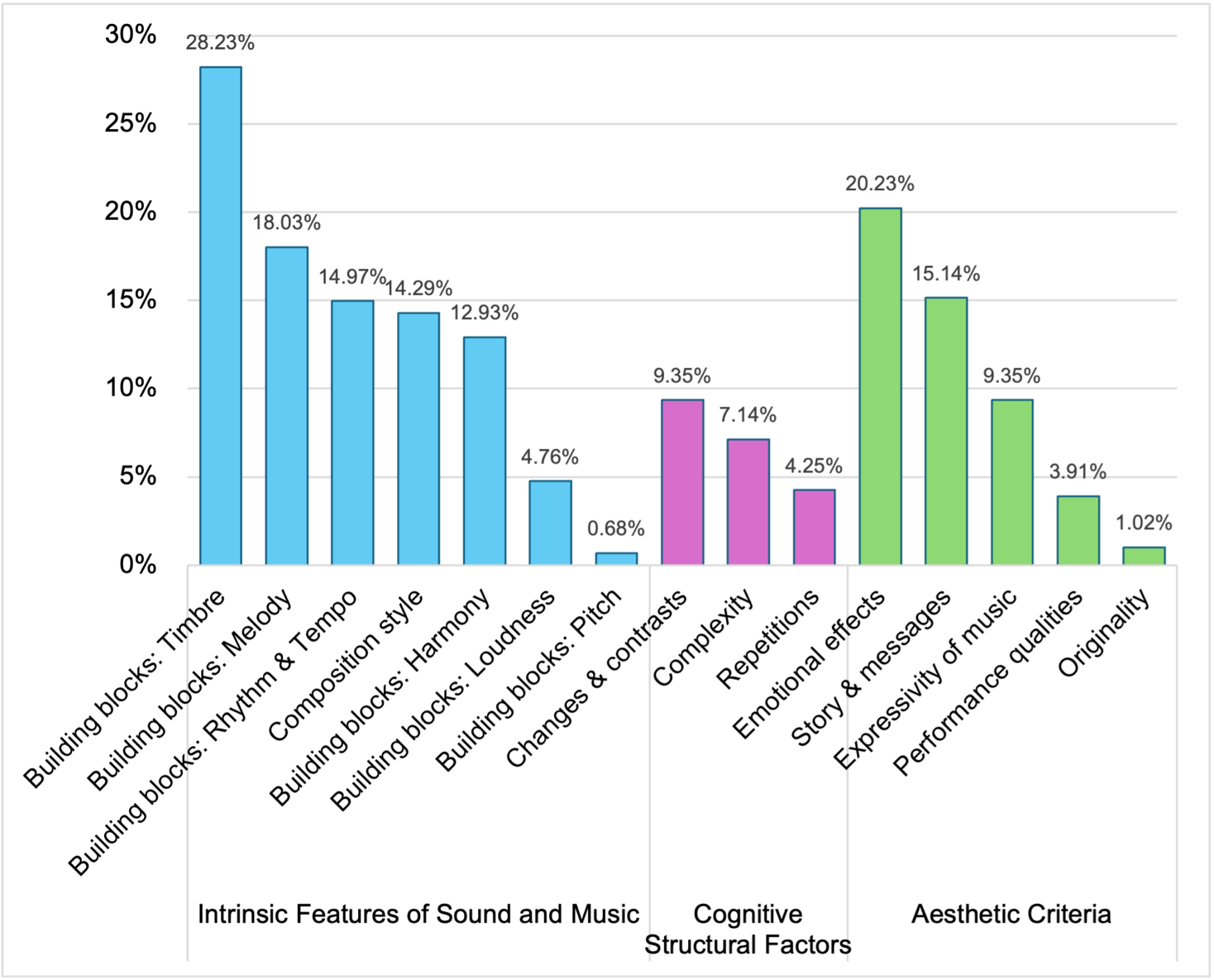
**

**Fig B. The rate of occurrence of each code: Affect**


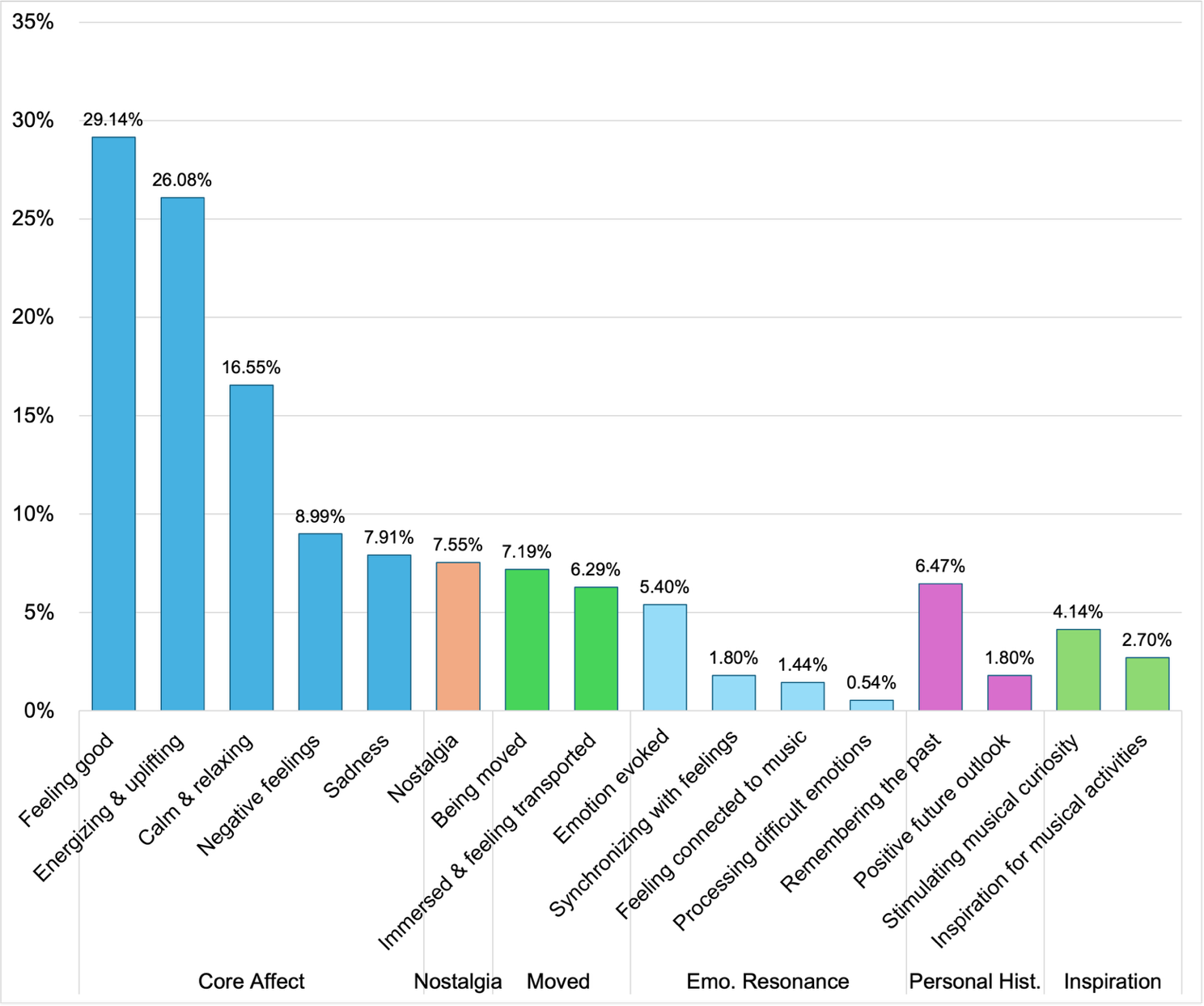


**Fig C. The rate of occurrence of each code: Impact**

**
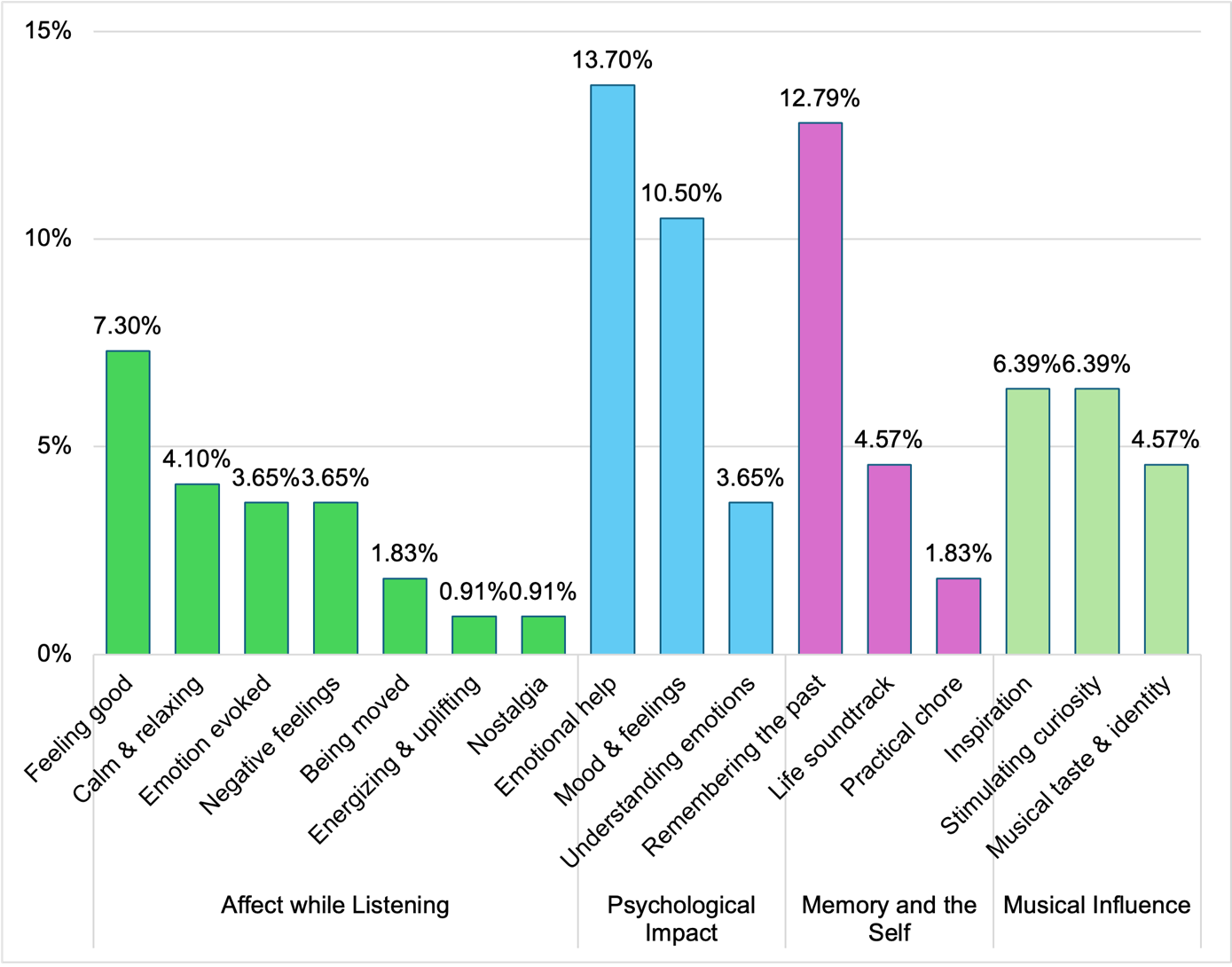
**
