# Supplementary material for: Unpacking musical beauty: Sound, emotion, and impact differences across expertise and personality
Source: PLoS One. 2025 Nov 14;20(11):e0335905. doi: 10.1371/journal.pone.0335905 (PMC12617921; doi:10.1371/journal.pone.0335905)
Supplement: S1 Appendix — (DOCX) [file pone.0335905.s002.docx]

**S2 Appendix: the list of pieces the participants reported as beautiful and not beautiful**

Tables A-O below show the titles and composers/artists of the pieces the participants listed as beautiful and not beautiful per genre. The genre of each piece was classified based on 14 genres proposed by Renfrow and Gosling (2003), using Spotify Web API (Spotify, n.d.) and the Spotify package in R (Thompson et al., 2022). We created a playlist of pieces mentioned by participants on Spotify and acquired information about the genre and audio features of each piece through Spotify Web API. Since Spotify Web API output lists many genres and sub-genres of music for each piece, we assigned whichever of the 14 genres from Renfrow and Gosling (2003) had the most mentions in Spotify Web API output. When Spotify Web API either did not return any output or returned the same number for more than one of the 14 genres, then genre classification was conducted manually by the first author and by two other music psychology researchers listening to each piece. Pieces that were judged as not belonging to any of 14 genres were classified as ‘other’.

Table A. Alternative

|  | **Beautiful** | | **Not beautiful** | |
| --- | --- | --- | --- | --- |
|  | **Title** | **Composer/artist** | **Title** | **Composer/artist** |
|  | Into My Arms | Nick Cave and The Bad Seeds | Cherub Rock | The Smashing Pumpkins |
|  | Living Hope | Phil Wickham | Me Gustas Tu | Manu Chao |
|  | Song for Zula | Phosphorescent | Rockstar | Nickelback |
|  | Yours | Evann McIntosh |  |  |
|  | You | Keaton Henson |  |  |
| **Total number**  **(percentage)** | 5 out of 230 (2.17%) | | 3 out of 228 (1.32%) | |

Table B. Blues

|  | **Beautiful** | | **Not beautiful** | |
| --- | --- | --- | --- | --- |
|  | **Title** | **Composer/artist** | **Title** | **Composer/artist** |
|  | I Believe I'll Dust My Broom | Robert Johnson | Short Change Hero | The Heavy |
| **Total number**  **(percentage)** | 1 out of 230 (0.43%) | | 1 out of 228 (0.44%) | |

Table C. Classical

|  | **Beautiful** | | **Not beautiful** | |
| --- | --- | --- | --- | --- |
|  | **Title** | **Composer/artist** | **Title** | **Composer/artist** |
|  | 10 Piano Pieces, Op. 12: V. Capriccio | Sergei Prokofiev | Air on the G String | Johann Sebastian Bach |
|  | 10 Preludes, Op. 23: No. 10 in G-Flat Major | Sergei Rachmaninoff | Canon in D Major | Johann Pachelbel |
|  | 8 Slavonic Dances, Op. 46, B.83: No. 8 in G minor, Presto | Antonín Dvořák | Concerto Grosso No.1 | Alfred Schnittke |
|  | Adagio for Strings, Op. 11 | Samuel Barber | Clair de lune, L.32 | Claude Debussy |
|  | Air on the G string | Johann Sebastian Bach | Electric Counterpoint: III. Fast | Steve Reich |
|  | An Die Musik | Franz Schubert | Étude in C Minor, Op. 10, No. 12: Allegro con fuoco | Frédéric Chopin |
|  | Ballade No. 1 in G Minor, Op. 23 | Frédéric Chopin | Firebird Suite | Igor Stravinsky |
|  | Bluebird | Alexis Ffrench | Gurrelieder | Gustav Mahler |
|  | Brandenburg Concerto No. 3 in G Major, BWV 1048: III. Allegro | Johann Sebastian Bach | Hoedown | Aaron Copland |
|  | Canon in D Major | Johann Pachelbel | Hungarian Dance No. 5 | Johannes Brahms |
|  | Cello Concerto in B Minor, Op. 104, B. 191: I. Allegro | Antonín Dvořák | Improvisations for Harp | William Mathias |
|  | Cello Concerto in E Minor, Op. 85: I. Adagio - Moderato | Edward Elgar | Introduction and Rondo capriccioso in A Minor, Op. 28 | Camille Saint-Saëns |
|  | Clair de Lune, L. 32 | Claude Debussy | Lulu | Alban Berg |
|  | Clarinet Concerto in A Major, K. 622: II. Adagio | Wolfgang Amadeus Mozart | Nocturne in E-flat Major, Op. 9, No.2 | Frédéric Chopin |
|  | Dido and Aeneas, “When I Am Laid in Earth” | Henry Purcell | Octandre | Edgard Varèse |
|  | Eine Kleine Nachtmusik | Wolfgang Amadeus Mozart | Octet in E-flat Major, Op. 20, MWV R20: I. Allegro moderato, ma con fuoco | Felix Mendelssohn |
|  |  |  |  |  |
| Table C. Classical (continued) | |  |  |  |
|  | **Beautiful** | | **Not beautiful** | |
|  | **Title** | **Composer/artist** | **Title** | **Composer/artist** |
|  | Fantasia on a Theme by Thomas Tallis | Ralph Vaughan Williams | Piano Concerto | Arnold Schoenberg |
|  | Farewell to Stromness | Peter Maxwell Davies | Piano Sonata No. 2 in D Minor, Op. 14: IV. Vivace | Sergei Prokofiev |
|  | Four Seasons | Antonio Vivaldi | Piano Sonata No.8 in C minor, op.31 | Ludwig van Beethoven |
|  | Für Elise | Ludwig van Beethoven | Prelude C-sharp minor | Sergei Rachmaninoff |
|  | Gnossienne: No. 1 | Erik Satie | Requiem | Giuseppe Verdi |
|  | Goldberg Variations, BWV 988: Aria | Johann Sebastian Bach | Rite of spring | Igor Stravinsky |
|  | Gymnopédie No. 1 | Erik Satie | S'Wonderful | George Gershwin |
|  | I Giorni | Ludovico Einaudi | Scherzando | Nikolai Rimsky-Korsakov |
|  | Jazz Suite No. 2: VI. Waltz 2 | Dmitri Shostakovich | Short Ride in a Fast Machine | John Adams |
|  | La Fille aux Cheveux de Lin, L. 33 | Claude Debussy | String Quartet No. 8 in C Minor, Op. 110: 3. Allegretto | Dmitri Shostakovich |
|  | [Les pêcheurs de perles,](https://en.wikipedia.org/wiki/Les_p%C3%AAcheurs_de_perles) "Au fond du temple saint" | Georges Bizet | String Quartet Op 59, No. 2 | Ludwig van Beethoven |
|  | Les Vêpres Siciliennes, Ballet of the Four Seasons: I. L'inverno | Giuseppe Verdi | Solomon, The Arrival of the Queen of Sheba | George Frideric Handel |
|  | Lieder ohne Worte, Op. 19: No. 4 in A (Moderato), MWV U 73 | Richard Wagner | Summertime | George Gershwin |
|  | Meditation | Jules Massenet | Symphonic Dances | Sergei Rachmaninoff |
|  | Melodie in E Major, Op. 3, No. 3 | Sergei Rachmaninoff | Symphonic Dances from West Side Story | Leonard Bernstein |
|  | Musica Incidental Campesina: I. Preludio | Leo Brouwer | Symphony No.5 | Ludwig van Beethoven |
|  |  |  |  |  |
| Table C. Classical (continued) | |  |  |  |
|  | **Beautiful** | | **Not beautiful** | |
|  | **Title** | **Composer/artist** | **Title** | **Composer/artist** |
|  | My First Homage | Gavin Bryars | Symphony No. 10 in E Minor, Op. 93: II. Allegro | Dmitri Shostakovich |
|  | Partita in D minor, BWV 1004: Chaconne | Johann Sebastian Bach | Swan Lake, Op. 20, Act 2: No. 10, Scene. Moderato | Pyotr Ilych Tchaikovsky |
|  | Peer Gynt, Op.23, Morning Mood | Edvard Greig | The Art of Fugue | Johann Sebastian Bach |
|  | Piano Concerto No. 1 in B-flat minor, Op. 23: I. Allegro non troppo e molto maestoso - Allegro con spirito | Pyotr Ilych Tchaikovsky | The Planets, Op. 32: I. Mars, the Bringer of War | Gustav Holst |
|  | Piano Sonata, No.14 in C-sharp minor, Op.27, No.2 | Ludwig van Beethoven | Trombone Concerto | Nikolai Rimsky-Korsakov |
|  | Piano Sonata No. 18 in D major, K.576: 1. Allegro | Wolfgang Amadeus Mozart | Turangalila symphony | Olivier Messiaen |
|  | Piano Sonata No. 8 in C minor, Op. 13 "Pathétique": II. Adagio cantabile | Ludwig van Beethoven | Violin Concerto, Op. 36 | Arnold Schoenberg |
|  | Prélude à l'après-midi d'un faune, L.86 | Claude Debussy | William Tell - Overture | Gioachino Rossini |
|  | Requiem in D minor, K. 626: Lacrimosa dies illa | Wolfgang Amadeus Mozart |  |  |
|  | Romeo and Juliet Fantasy Overture, TH 42 | Pyotr Ilych Tchaikovsky |  |  |
|  | Salut d'Amour, Op. 12 | Edward Elgar |  |  |
|  | Scherzo No. 2 in B-flat minor, Op. 31 | Frédéric Chopin |  |  |
|  | Second Prelude | George Gershwin |  |  |
|  | Spartacus Suite No. 2: Adagio of Spartacus and Phrygia | Aram Khachaturian |  |  |
|  | String Sextet No 1, Op 18: II | Johannes Brahms |  |  |
|  | Suite No. 3 in D Major, BWV 1068: 2. Air | Johann Sebastian Bach |  |  |
|  |  |  |  |  |
| Table C. Classical (continued) | |  |  |  |
|  | **Beautiful** |  | **Not beautiful** |  |
|  | **Title** | **Composer/artist** | **Title** | **Composer/artist** |
|  | Symphony No. 2 in E minor, Op. 27: III. Adagio | Sergei Rachmaninoff |  |  |
|  | Symphony No. 3 in F Major, Op. 90: III. Poco allegretto | Johannes Brahms |  |  |
|  | Symphony No. 5 | Ludwig van Beethoven |  |  |
|  | Symphony No. 5: II. stürmisch bewegt, mit grösster Vehemenz | Gustav Mahler |  |  |
|  | Symphony No. 8 in G minor, Op. 88, B. 163: I. Allegro con brio | Antonín Dvořák |  |  |
|  | The Armed Man - A Mass for Peace: XII. Benedictus | Karl Jenkins |  |  |
|  | The Four Seasons, Violin Concerto in E Major, Op. 8 No. 1, RV 269 "Spring": I. Allegro | Antonio Vivaldi |  |  |
|  | The Lark | Mikhail Glinka |  |  |
|  | The Morrow | Michael Nyman |  |  |
|  | The Planets Suite, Op. 32: IV. Jupiter | Gustav Holst |  |  |
|  | Triple Concerto in C Major, Op. 56: II. Largo | Ludwig van Beethoven |  |  |
|  | Verklärte Nacht, Op. 4 | Arnold Schoenberg |  |  |
|  | Vier letzte Lieder, No. 3 | Richard Strauss |  |  |
|  | Violin Concerto No. 5 in A Major, K. 219 "Turkish": I. Allegro aperto | Wolfgang Amadeus Mozart |  |  |
|  | Waltz in A Minor, Op. Posth., B. 150 | Frédéric Chopin |  |  |
|  | White Landscapes, Op. 47a: III. Disappearance of Snow. Largo | Takashi Yoshimatsu |  |  |
| **Total number (percentage)** | 64 out of 230 (27.83%) | | 40 out of 228 (17.54%) | |

Table D. Country

|  | **Beautiful** | | **Not beautiful** | |
| --- | --- | --- | --- | --- |
|  | **Title** | **Composer/artist** | **Title** | **Composer/artist** |
|  | Hurt | Trent Reznor/Johnny Cash | Before He Cheats | Carrie Underwood |
|  | Wichita Lineman | Glen Campbell |  |  |
| **Total number (percentage)** | 2 out of 230 (0.78%) | | 1 out of 228 (0.44%) | |

Table E. Electronica/dance

|  | **Beautiful** | | **Not beautiful** | |
| --- | --- | --- | --- | --- |
|  | **Title** | **Composer/artist** | **Title** | **Composer/artist** |
|  | Break My Heart | Rudimental | Baddadan | Chase & Status |
|  | Calypso | Lvte Bloomer | Cadence - VIP | Metrik |
|  | Dream Getaway | Maximum Love | Canned Heat | Jamiroquai |
|  | Fade Away | Yula | Empire Of Steel | Essenger |
|  | Gemini | Gemini & George Maple | Empty | Empty |
|  | Kolfax | O'Flynn & Frazer Ray | Fools Gold | The Stone Roses |
|  | Music Sounds Better With You | Stardust | Glue | Glue |
|  | Somebody Like U | Alan Walker | I Don't Feel Like Dancin' | Scissor Sisters |
|  | Strings Again | Matisse & Sadko | Join Us for a Bite | The Living Tombstone |
|  | Voyager | Daft Punk | Offender | Dimension & Grafix |
|  |  |  | Piezo | Piezo |
|  |  |  | Set Me Free | Friction |
|  |  |  | Chto-to Osoboe Vo Mne | Shortparis |
|  |  |  | My Head Feels Like A Moshpit | Just Reality & Saleum |
| **Total number (percentage)** | 10 out of 230 (4.35%) | | 14 out of 228 (6.14%) | |

Table F. Folk

|  | **Beautiful** | | **Not beautiful** | |
| --- | --- | --- | --- | --- |
|  | **Title** | **Composer/artist** | **Title** | **Composer/artist** |
|  | A Case of You | Joni Mitchell | Huginn & Munin | Ten Strings and a Goat skin |
|  | Anything | Adrianne Lenker | Rain Dogs | Tom Waits |
|  | Blackbird | Traditional/Martyn Bennett | Save the Bees | Lau |
|  | Ca' The Yowes | Robert Burns/Traditional | Shades and Shadows | Peter Gundry |
|  | Canyon Moonrise | John McGann | The Death of the Dining Car | Lau |
|  | Considerate Birders | George Sansome | Wild Mountainside | Eddi Reader |
|  | His Friends Are More Than Fond of Robin | Carly Simon |  |  |
|  | Over The Rainbow | Eva Cassidy |  |  |
|  | The Kiss | Judee Sill |  |  |
|  | The Town I Loved So Well | Phil Coulter |  |  |
|  | The Weakness in Me | Joan Armitrading |  |  |
|  | To Build A Home | Cinematic Orchestra |  |  |
|  | Unquiet Grave | Traditional/Lau |  |  |
|  | Candles | Daughter |  |  |
| **Total number (percentage)** | 14 out of 230 (6.09%) | | 6 out of 228 (2.63%) | |

Table G. Heavy metal

|  | **Beautiful** | | **Not beautiful** | |
| --- | --- | --- | --- | --- |
|  | **Title** | **Composer/artist** | **Title** | **Composer/artist** |
|  | Changes | Ozzy Osbourne | Angel Of Death | Angel Of Death |
|  | Cradle Song | Iamthemorning | From Nothing | Reflections |
|  | Ice Fist | Vampillia | Hallowed Be Thy Name | Iron Maiden |
|  | Sextape | Deftones | Let It Roar | Battle Beast |
|  |  |  | Master Of Puppets | Metallica |
|  |  |  | My Name Is Mud | Primus |
|  |  |  | New Modern Love | Halestorm |
|  |  |  | Raining Blood | Slayer |
|  |  |  | Sober | Sober |
|  |  |  |  |  |
|  |  |  |  |  |
| Table G. Heavy metal (Continued) | |  |  |  |
|  | **Beautiful** | | **Not beautiful** | |
|  | **Title** | **Composer/artist** | **Title** | **Composer/artist** |
|  |  |  | Stinkfist | Tool |
|  |  |  | The Broken Cross | Distant |
|  |  |  | The Trooper | Iron Maiden |
|  |  |  | Wait And Bleed | Slipknot |
|  |  |  | War Pigs | Black Sabbath |
| **Total number (percentage)** | 4 out of 230 (1.74%) | | 14 out of 228 (6.14%) | |

Table H. Jazz

|  | **Beautiful** | | **Not beautiful** | |
| --- | --- | --- | --- | --- |
|  | **Title** | **Composer/artist** | **Title** | **Composer/artist** |
|  | Better Git It in Your Soul | Charles Mingus | Judas | Esperanza Spalding |
|  | Bring It on Home to Me | Roy Hargrove | Sway | Dean Martin |
|  | Day 5: For Carol | Tom Misch | Tenor Madness | Sonny Rollins |
|  | Face in the Crowd | Kansas Smittys | The Girl from Ipanema | Astrud Gilberto |
|  | Isa Lei | Ry Cooder and Vishwa Mohan Bhatt |  |  |
|  | Lingus (We Like It Here) | Snarky Puppy |  |  |
|  | O Tannenbaum | Vince Guaraldi trio |  |  |
|  | Saint James Infirmary Blues | Jon Batiste |  |  |
|  | Symbiosis, 2nd Movement: Largo - Andante - Maestoso - Largo, Pt.1 | Bill Evans |  |  |
|  | Valentine | Laufey |  |  |
|  | Kind Of Blue | Miles Davids |  |  |
| **Total number (percentage)** | 11 out of 230 (4.78%) | | 4 out of 228 (1.75%) | |

Table I. Pop

|  | **Beautiful** | | **Not beautiful** | |
| --- | --- | --- | --- | --- |
|  | **Title** | **Composer/artist** | **Title** | **Composer/artist** |
|  | 7 Years | Lukas Graham | 2020 | Suuns |
|  | Another Love | Tome Odell | Alive | Pearl Jam |
|  | Bitter Sweet Symphony | The Verve | All I Want for Christmas Is You | Mariah Carey |
|  | Bless The Telephone | Labi Siffre | Army Of Me | Bjork |
|  | Chun-li | Nicki Minaj | Asimbonanga (Mandela) | Johnny Clegg |
|  | Double Take | Dhruv | Bassline | Reverand And the Makers |
|  | Emotions | Mariah Carey | Build Me Up Buttercup | Foundations |
|  | Forever Young | Alphaville | Clocks | Coldplay |
|  | Francesca | Hozier | Cupid | Fifty Fifty |
|  | Hallelujah | Jeff Buckley | Dancing Queen | Abba |
|  | Hallelujah | Pentatonix | Dial Drunk | Noah Kahan |
|  | Heaven | Avicii | Every Teardrop Is a Waterfall | Coldplay |
|  | Hope There's Someone | Anthony And the Johnsons | Gatti | Jackboys & Travis Scott |
|  | Hung Up | Madonna | Gats | Susumu Hirasawa |
|  | I Know the End | Phoebe Bridgers | Getting Away with It (All Messed Up) | James |
|  | Let Her Go | Mike Rosenberg | Happier | Ed Sheeran |
|  | Moments Of Pleasure | Kate Bush | How You Like That | Blackpink |
|  | Never Let Her Slip Away | Andrew Gold | Hung Up | Madonna |
|  | Not Strong Enough | Boygenius | I Feel Love | Donna Summer |
|  | Nova | VNV Nation | I'll Be There for You | Redbrandts |
|  | O | Coldplay | I'm Just a Kid | Simple Plan |
|  | Perfect | Ed Sheeran | Levitating | Dua Lipa |
|  | Pizza | Martin Garrix | Low | Flo Rida |
|  | Praying For Time | George Michael | M.I.L.F. $ | Fergie |
|  | Pyjama Pants | Cavetown | Man's World | Marina |
|  | Roads | Portishead | My Happy Ending | Avril Lavigne |
|  | Rock & Roll I Gave You the Best Years of My Life | Kevin Johnson | Mythological Beauty | Big Thief |
|  | Shadows | Lindsey Stirling | Northern Attitude | Noah Kahan |
| Table I. Pop (Continued) | |  |  |  |
|  | **Beautiful** | | **Not beautiful** | |
|  | **Title** | **Composer/artist** | **Title** | **Composer/artist** |
|  | Something Just Like This | Coldplay | Orange Trees | Marina |
|  | Stick Season | Noah Kahan | Paint My Bedroom Black | Holly Humberstone |
|  | Still Life | Still Life | Rocks At My Window | Bridgit Mendler |
|  | Stuck On You | Lionel Ritchie | September Song | Agnes Obel |
|  | The Power of Love | Celine Dion | Sisyphus | Andrew Bird |
|  | The Riddle | Nik Kershaw | So Hot You're Hurting My Feelings | Caroline Polachek |
|  | The Scientist (Glee Cast Version) | Glee Cast | Stronger | Britney Spears |
|  | The Sound of Silence | Pentatonix | Sur Le Pavé | Holden |
|  | Thinking Out Loud | Ed Sheeran | The Man | Taylor Swift |
|  | This Woman's Work | Kate Bush | The Middle | Jimmy Eat World |
|  | Thumbs | Lucy Dacus | The Power of Love | Celine Dion |
|  | When I Look at You | Miley Sirus | This Is What They Meant | Big Piig |
|  | Cake | Melanie Martinez | Umbrella | Rihanna |
|  | Champagne Problems | Taylor Swift | You'll Miss Me When I'm Not Around | Grimes |
|  | Long Story Short | Taylor Swift | You're Moving Out Today | Carole Bayer Sager |
|  | Tolerate It | Taylor Swift | Bad Idea Right? | Olivia Rodrigo |
|  |  |  | Beautiful | Christina Agulera |
|  |  |  | Cash Machine | Oliver Tree |
|  |  |  | Walking Away | Craig David |
| **Total number (percentage)** | 44 out of 230 (19.13%) | | 47 out of 228 (20.61%) | |

Table J. Rap/hiphop

|  | **Beautiful** | | **Not beautiful** | |
| --- | --- | --- | --- | --- |
|  | **Title** | **Composer/artist** | **Title** | **Composer/artist** |
|  | Drunk In Love | Beyonce | 6 Kiss | YNW Melly & Jamell Maurice Demons |
|  | Humble. | Kendrick Lamar | C'mon (Catch 'Em By Surprise) | Busta Rhymes Et Al |
|  | One Dance | Drake Ft Wizkid | Dui | Lil Tecca |
|  | Temptation | Joey Bada$$ | Lightskin Lil Wayne | Tyga |
|  |  |  | Meltdown | Travis Scott Feat. Drake |
|  |  |  | No Worries | Loyle Carner |
|  |  |  | Put It On | Big L |
|  |  |  | Rago | A Star |
|  |  |  | Rap God | Eminem |
|  |  |  | Runnin' | Pharcyde |
|  |  |  | Shake That | Eminem |
|  |  |  | Special | Lizzo |
|  |  |  | Started Again | Triple O |
|  |  |  | Swimming Pools (Drank) | Kendrick Lamar |
|  |  |  | The Box | Rody Ricch |
|  |  |  | Toxic (Freestyle) | Songer |
|  |  |  | Unforgettable | French Montana |
|  |  |  | Wandered To LA | Juice WRLD |
| **Total number (percentage)** | 4 out of 230 (1.74%) | | 18 out of 228 (7.89%) | |

Table K. Religious

|  | **Beautiful** | | **Not beautiful** | |
| --- | --- | --- | --- | --- |
|  | **Title** | **Composer/artist** | **Title** | **Composer/artist** |
|  | Cast your Cares | Guvna B |  |  |
|  | Merciful God | Daphne Richardson |  |  |
|  | Ubi Caritas | Durufle |  |  |
| **Total number (percentage)** | 3 out of 230 (1.90%) | | 0 out of 228 (0%) | |

Table L. Rock

|  | **Beautiful** | | **Not beautiful** | |
| --- | --- | --- | --- | --- |
|  | **Title** | **Composer/artist** | **Title** | **Composer/artist** |
|  | A Wave Across a Bay | Frank Turner | Animal | Def Leppard |
|  | Albatross | Fleetwood Mac | Back In Black | AC/DC |
|  | Because | The Beatles | Bat out of Hell | Meatloaf |
|  | Between the Bars | Elliot Smith | Blood In the Water | Normandie |
|  | God Only Knows | Brian Wilson / Beach Boys | Bohemian Rhapsody | Queen |
|  | I Wanna Be Yours | Arctic Monkeys | Closer | Closer |
|  | Jesus Christ 2005 God Bless America | The 1975, Phoebe Bridgers | Come Alive | Foo Fighters |
|  | Landslide | Fleetwood Mac | Creep | Radiohead |
|  | Let Down | Radiohead | Die For You | Starset |
|  | Love Of My Life | Queen | Debaser | Pixies |
|  | Mind Games | John Lennon | Deeper High | Novadriver |
|  | Nights In White Satin | Moody Blues | Do I Wanna Know? | Arctic Monkeys |
|  | Nude | Radiohead | Don't Stop Me Now | Queen |
|  | Seasons | Chris Cornell | E-Bow The Letter | R.E.M. |
|  | Streets Of Philadelphia | Bruce Springsteen | Electric Love | Borns |
|  | The Sound Of Silence | Disturbed | Friday On My Mind | The Easybeats |
|  | The Stolen Child | Waterboys | Give It Away | Red Hot Chili Peppers |
|  | Tubular Bells - Pt. I | Mike Oldfield | Good As It Gets | Anti-nowhere League |
|  | Waterloo Sunset | The Kinks | Heat Above | Greta Van Fleet |
|  | Weird Fishes | Radiohead | Heroes | David Bowie |
|  | Wish You Were Here | Pink Floyd | How Am I Supposed To Live Without You | Michael Bolton |
|  | You Are The Everything | R.E.M. | Kickstart My Heart | Motley Crue |
|  | Zombie | The Cranberries | Killer Queen | Queen |
|  | About A Gitl | Nirvana | Loser | Beck |
|  | Come As You Are | Nirvana | Love Like Revenge | Bad Suns |
|  |  |  | Marquee Moon | Television |
|  |  |  | Monkberry Moon Delight | Paul Mccartney |
|  |  |  | Mr. Brightside | The Killers |
|  |  |  | Mutt | Blink 182 |
|  |  |  | My Lovely Man | Red Hot Chili Peppers |
|  |  |  | N.I.B. | Black Sabbath |
|  |  |  | Natural | Imagine Dragons |
|  |  |  |  |  |
| Table L. Rock (Continued) | |  |  |  |
|  | **Beautiful** | | **Not beautiful** | |
|  | **Title** | **Composer/artist** | **Title** | **Composer/artist** |
|  |  |  | Out Of The Frying Pan (And Into The Fire) | Meatloaf |
|  |  |  | Paperback Writer | Beatles |
|  |  |  | Pitseleh | Elliot Smith |
|  |  |  | Radio Ga Ga | Queen |
|  |  |  | Reptilia | The Strokes |
|  |  |  | Rocket Man (I Think It's Going To Be A Long, Long Time) | Elton John |
|  |  |  | Saturday Night Special | Lynryd Skynryd |
|  |  |  | Seed | Sublime |
|  |  |  | Start Me Up | Rolling Stones |
|  |  |  | The Hero !!: Ikareru Kobushi Ni Hi O Tsukero | JAM Project |
|  |  |  | Teenage Kicks | The Undertones |
|  |  |  | The Losing Side | Grace Petrie |
|  |  |  | The National Anthem | Radiohead |
|  |  |  | The Sound Of Silence | Simon And Garfunkel |
|  |  |  | Touch Too Much | AC/DC |
|  |  |  | Tubular Bells | Mike Oldfield |
|  |  |  | Two Fingers | Jake Bugg |
|  |  |  | Unsung Hero | Area 7 |
|  |  |  | Vertigo | U2 |
|  |  |  | W.O.L.D. | Harry Chapin |
|  |  |  | Waxie's Dargle | The Pogues |
|  |  |  | Welcome To The Black Parade | My Chemical Roamance |
|  |  |  | Wide Awake | Audioslave |
|  |  |  | Working On The Highway | Bruce Springsteen |
|  |  |  | Young Folks | Peter Bjorn And John |
|  |  |  | Body | Mother Mother |
| **Total number (percentage)** | 25 out of 230 (10.87%) | | 57 out of 228 (25.00%) | |

Table M. Soul/funk

|  | **Beautiful** | | **Not beautiful** | |
| --- | --- | --- | --- | --- |
|  | **Title** | **Composer/artist** | **Title** | **Composer/artist** |
|  | Green Onions | Booker T. & the MG's | Ain't No Way | Aretha Franklin |
|  | October | Eric Whitacre | Babydoll | Ari Abdul |
|  | Strange | Celeste | September | Earth wind and fire |
|  | Heal the World | Micheal Jackson | Snake Style 2 | Snake Style |
|  | Open Up | UMI | Under the influence | Chris Brown |
|  |  |  | Whatever U Did | Annix |
|  |  |  | Heal the World | Micheal Jackson |
| **Total number (percentage)** | 5 out of 230 (2.17%) | | 7 out of 228 (3.07%) | |

Table N. Sound track

|  | **Beautiful** | | **Not beautiful** | |
| --- | --- | --- | --- | --- |
|  | **Title** | **Composer/artist** | **Title** | **Composer/artist** |
|  | Do This Thing | Cast of Mean Girls on Broadway | Battle Urge | Kenichiro Suehiro |
|  | Flying | John Williams | Summer 78 (1) (2) | Yann Tiersen |
|  | Jacob and the Stone | Emile Mosseri | The Archers | Arthur Wood |
|  | My Octopus Teacher End Credits | kevin smuts | Thomas and the Friends Theme | Mike O'Donnell and Junior Campbell |
|  | One Summer Day | Joe Hisaishi |  |  |
|  | Say It Somehow | Adam Guettel |  |  |
|  | Still Life at the Penguin Cafe | Penguin Cafe Orchestra |  |  |
|  | A Drop Filled With Memories | Susumu Hirasawa |  |  |
|  | The Ecstasy of Gold | Ennio Morricone |  |  |
|  | The Return of the King | Howard Shore |  |  |
|  | Theme From Schindler's List | John Williams |  |  |
|  | Time | Hans Zimmer |  |  |
|  | Unchained Melody | Maurice Jarre |  |  |
|  | Vide Cor Meum | Patrick Cassidy |  |  |
| **Total number (percentage)** | 14 out of 230 (6.09%) | | 4 out of 228 (1.75%) | |

Table O. Other

|  | **Beautiful** | | **Not beautiful** | |
| --- | --- | --- | --- | --- |
|  | **Title** | **Composer/artist** | **Title** | **Composer/artist** |
|  | All The Earth | Jessica Curry | Calm Spirit | Roal Marquis |
|  | Bonde | Ali Farka Toure | Do You Hear the People Sing? | Claude-Michel Schonberg |
|  | Cornfield Chase | Dorian Marko | Helmet | Steve Lacy |
|  | Experience | Einaudi | Opening: The New World | Songs for a new world |
|  | Flight from the City | Johan Johannsonn | Organise | Asake |
|  | Flora | Hiroshi Yoshimura | Peaceful journey | Anna Vince |
|  | JTEL | Blithe Field | Sleeping beauty Lullaby | Enno Aare |
|  | Mambo Sinuendo | Ry Colder, Manuel Galban | Wheels on the Bus | Nursery Rhyme |
|  | New Life | Thomas Bergersen | Calm Down | REMA |
|  | Nuvole Bianche | Einaudi | Jireh (My Provider) | Lecrae |
|  | Queremos Paz | Gotan Project | Lonely At the Top | Asake |
|  | River Flows in You | Yiruma | Unavailable | Davido |
|  | Seven | Andrea Casarrubious |  |  |
|  | Una Mattina | Ludovico Einaudi |  |  |
|  | What Was Said | Ashley Bathgate, Paul Fowler |  |  |
|  | Lonely Waltz | Ophelia Wilde |  |  |
|  | Saturn | Sleeping At Last |  |  |
|  | Edelweiss | Rogers/Hammerstein |  |  |
|  | Elm | Martin Landh |  |  |
|  | Hold On Me | Called Out Music |  |  |
|  | Plus Tã´t | Alexandra Streliski |  |  |
|  | Says | Nils Frahm |  |  |
|  | You Are My Home | GB 0:07 / 3:18 Thomas Bergersen |  |  |
|  | No Woman No Cry | Bob Marley |  |  |
| **Total number (percentage)** | 24 out of 230 (10.43%) | | 12 out of 228 (5.26%) | |

**References**

Rentfrow, P. J., & Gosling, S. D. (2003). The do re mi's of everyday life: The structure and personality correlates of music preferences. *Journal of Personality and Social Psychology*, *84*(6), 1236-1256. doi.org/10.1037/0022-3514.84.6.1236

Thompson, C., Parry, J., Phipps, D., & Wolff, T. (2022, December 15). R Wrapper for the ‘Spotify’ Web API. https://www.rcharlie.com/spotifyr/
