# Supplementary material for: Unpacking musical beauty: Sound, emotion, and impact differences across expertise and personality
Source: PLoS One. 2025 Nov 14;20(11):e0335905. doi: 10.1371/journal.pone.0335905 (PMC12617921; doi:10.1371/journal.pone.0335905)
Supplement: Attachment — Submitted filename: Response_Letter.docx [file pone.0335905.s004.docx]

We would like to thank the reviewers for their valuable and constructive comments and helpful suggestions. We truly appreciate the time and effort invested in reviewing this manuscript. We have carefully considered all suggestions and comments, and revised the manuscript accordingly. The detailed responses to each comments are provided below.

**Reviewer #1:**

I would like to thank author(s) for the opportunity to review this manuscript. The paper addresses an interesting topic.
This study examined how individuals experience musical beauty by comparing self-selected beautiful and non-beautiful pieces. Analysis of responses revealed that beautiful music is characterized by gentle acoustic features and evokes emotions, such as calmness and pleasant sadness. Beauty evoked by music also provides emotional support and inspiration. Listeners with highly openness were especially sensitive to musical aesthetics, highlighting the role of personality in shaping beauty perception.
However, several points would benefit from further clarification or revision to strengthen the overall quality and impact of the manuscript.
Three points are particularly concerned.
1. The novelty of the research has not been shown.
2. The introduction and discussion do not explain sufficiently most relevant studies that are similar to this study. In the introduction and discussion, although extensive references are provided, they do not appear to be organically integrated with the aim and interpretation of results of this study.
3. Redundancy of descriptions and obscurity of the definition of beauty
The results are presented in a lengthy manner because the elements obtained from the participants' responses are listed in order, which can make them appear redundant. The results are explained using various insights such as emotions and nostalgia, but it is somewhat unclear whether they can truly be explained as “beauty.”

For details, please see below.

*Thank you very much for pointing out these three points.*

*1. We have now worked carefully to clarify the study’s novelty throughout the manuscript.*

*2. All reviewed literature (apart from the earliest context-setting opening comments) now directly and explicitly motivate the gaps we have identified in the introduction, and we now very explicitly contextualize all findings in our Discussion section.*

*3. We have carefully revisited the results to make them more concise and logically presented.*

*The details of changes made can be seen under each comment.*

**Comment#1**: Introduction
p.3, l.49-52: This is an overstate, given many psychological studies on music and beauty. To avoid misunderstanding by readers, a more modest expression should be used. In particular, the following papers appear to be related to this study. Fleckenstein et al. (2025) is particularly similar in terms of methodology. This study should discuss previous studies sufficiently and clarify what is known and what is unknown in order to describe the research gap. This will make it easier for readers to understand the significance of this study.
Furthermore, it should be comparable to the latest research by Brattico et al. (2025) using listening experiments. If this study delves into subjective perceptions of beauty using a method different from listening experiments, explaining the similarities and differences with actual listening will help readers gain a deeper understanding.

Fleckenstein, A. M., Vuoskoski, J. K., & Dibben, N. (2025). Understanding Musical Beauty. Empirical Studies of the Arts, 43(1), 505-523.

Brattico, E., Brusa, A., Dietz, M., Jacobsen, T., Fernandes, H. M., Gaggero, G., ... & Proverbio, A. M. (2025). Beauty and the brain–Investigating the neural and musical attributes of beauty during naturalistic music listening. Neuroscience, 567, 308-325.

*Thank you for your comments. We agree that we may have misrepresented the volume of recent work that has been done in this area. We also agree that these two studies may be two of the more similar papers to ours with respect to the aims and the questions addressed. We now describe them in the necessary detail in the introduction, clarifying where remaining research gaps lie and how our study addresses these research gaps. Importantly, we now also provide more details about the many other relevant studies: this, so as to more clearly demonstrate the significance of the current study.*

*Specifically, firstly, instead of saying ‘at least from a psychological point of view, the experience of musical beauty remains largely unexplored’, we say that:*

-Line 51-52, page 3

‘research on musical beauty from a psychological perspective is still relatively limited’

*Secondly, we have highlighted Fleckenstein et al. (2024) ‘s paper as below*

Line 74, page 4

‘Recent studies have used wide-ranging methods to tackle some of the fundamental questions regarding musical beauty. One study by Fleckenstein et al [12] was particularly valuable in exploring participants’ subjective definition of musical beauty. Their study revealed that participants mainly view musical beauty as both an emotional experience on the one hand and as determined by the properties of an object on the other. The study also identified various factors that contribute to the experience of musical beauty, including participants' felt emotional experiences, the emotions expressed by the music, the timbre of instruments, and the memories listeners associated with the music.’

*Thirdly, we have highlighted Brattico et al. (2025) ‘s paper as below*

-Line 81, p.4

‘Complementing the qualitative approach taken in that study, Brattico et al [10] investigated the neural correlates of the experience of musical beauty, differentially associating different structures (orbitofrontal activity and bilateral supratemporal activity) with the experience of beauty. That study also revealed differences in sonic features of beautiful and not beautiful sections of music whereby beautiful, compared to not beautiful, sections were rated (by composers) as more tonal, simple, melodic, traditional, calm and sad.’

**Comment#2**: Introduction Features of music perceived as beautiful
p.4, l.75-76 ‘chills, being moved and the feeling of awe’
It is necessary to explain to readers that chills or being moved are appropriate examples of aesthetic experiences. In many cases, these are often considered in relation to emotion. To avoid such confusion, it can be better to describe (or operationally define) what constitutes an aesthetic experience.

*Thank you for this point. However, we have now removed the reference to aesthetic experience in this sentence as we realized it was making the sentence unnecessarily complicated.*

*We have nevertheless introduced the definition of an aesthetic judgment later on when referring to the literature that uses that term.*

-Line 67. page 3

‘An aesthetic judgement is considered an output, along with emotion and preferences, of the processing of artworks and other objects. This form of processing is held to involve perceptual, cognitive, and affective components, e.g., [21,25,26]’

**Comment #3**: Introduction
p.4, l.79-82: Prior research should be explained thoroughly. Rather than just referring to Omigie et al. [29] in detail, the author(s) should summarize all the studies mentioned in this section concisely, explaining what has been clarified and what has not been clarified in the previous studies cited.

*Thank you. We agree. This revised paragraph now concisely summarises many relevant studies and clarifies the research gap.*

-Line 108-127, page 5-6

‘The extent to which specific sonic features might be tied to key experiences of music (such as chills, being moved and the feeling of awe) has seen significant interest in the music psychology literature [30–33]. These studies have aligned in highlighting the role of both lower-level musical elements (such as loudness, tempo, and timbre) and higher-order, structural characteristics (such as tonal clarity) in affording these experiences. Regarding musical beauty, consonance—two or more harmoniously sounding concurrent tones—has been traditionally associated with beauty in the context of Western tonal music [34,35]. More recently, Fleckenstein et al.’s [12] participants reported not just harmonic content, but also instrumental timbre and tempo/rhythm/meter as factors contributing to their experience of musical beauty.

Interestingly, studies which have examined sections of pieces considered beautiful by listeners highlight the potential importance of temporal changes in musical elements for the experience of musical beauty. Beautiful sections in music, compared to not beautiful sections have been associated with more tonal, simple, melodic, traditional features [10], raising the possibility that listeners may find these moments more beautiful due to how they contrast with other moments. Similarly, a study by Omigie et al [13] demonstrated that while beautiful passages or sections of music could can vary in characteristics, they are often characterised by change such as a drop in tempo and a reduction in polyphony in some cases, or increases in dynamics, pitch register, major tonality, and harmonic clarity, in others.’

**Comment#4**: Introduction
p.5, l.95-97: Adding a clear explanation of the relationship between beauty and processing fluency and musical knowledge will make it easier for readers to understand.

*Thank you for pointing this out. We have now rephrased this sentence as follows;*

-Line 130, page 6

‘Indeed, according to the processing fluency theory [38], perceived beauty may be influenced by the ease with which a piece can be cognitively processed, where the easier it is to process the piece, the more beauty may be experienced.’

**Comment #5**: Feelings and emotion when experiencing beauty in music
p.6, l.122-123: ‘That perceived beauty and sadness in music are positively correlated [50,52]…’ These studies suggest that sad music can induce positive emotions. If so, simply saying “sadness” is insufficient, and a more specific explanation is preferable.

*Apologies that we were not clear. Whilst these studies showed positive relationships between beauty and sadness (hence our statement), we have now revised the sentence to make that clearer, and to emphasise further reasons (including the role of epistemic emotions) why beauty should be considered multifaceted and going beyond positive valance.*

-Line 157, page 7

‘This research shows that, alongside positive valence or pleasure, experiencing beauty from artworks is often linked with the feeling of being moved [33,49,50], the feeling of positive awe [32], and the feeling of sadness [51–54]. Further, hinting at the additional role that epistemic emotions may play, Armstrong and Detweiler-Badell [47] argue that the desire to understand a piece and the satisfactory resolution of such desire through apprehending the piece, are essential for experiencing beauty.

The variety of emotions associated with beauty experiences so far suggest that characterising beauty as pleasure alone may not fully capture its multifacetedness, compared to other forms of pleasure [46]. However, in light of ongoing debates about the relevance of distinguishing aesthetic pleasure from other forms [46], systematic research into the emotions that accompany beauty experiences may be considered increasingly necessary.’

Comment #6: Individual differences: musical training and personality
p.8, l.174-177: This assumption is probably too definitive. Musicians are expected to be more involved in music. In other words, they are likely to spend more time engaging in music and thinking about it. This will increase their exposure to musical beauty.

*Thank you for this comment. We initially meant to suggest that musicians may have been more impacted by beauty in the past and that is the reason why they choose their profession. However, we agree that the direction of causality could go both ways, since it is also possible that those who choose to be a musician may consequently then experience more impact from music.*

*We have now clarified these alternative possibilities while pointing out that both would nevertheless be reflected in a positive relationship between the musicianship and reported impact of beautiful music.*

-Line 218, page 10.

‘Further, since it has been reported that people who pursue music professionally often had strong emotional and aesthetic experiences of music during childhood [64,65], or a strong sense of calling for music during adolescence [66], one could predict that musicians, more than non-musicians, will report having been more impacted by musical beauty over the course of their lives. Here, it is important to note that being a musician may provide more opportunities to experience the possible impacts of music, and that as such impacts reported this group may be higher for that reason. In either case, a positive relationship between musicianship and breadth or scale of impacts from beautiful music seems a reasonable prediction to make.’

**Comment #7**: Individual differences: musical training and personality
p.9, l.198-200: Based on the previous research, it is necessary to discuss what further consideration is required. This is because the previous research summarized in this paragraph appears to have already been considered to a certain extent.

*Thank you for mentioning this. We have now revised this paragraph to clarify that what further consideration is required;*

-Line 247, page 11

‘Taken together, the findings from previous studies point to the possibility that musical beauty may subjectively differ according to the listener’s musical training and personality traits. Musicianship and Openness to experience may influence the ability to discuss features contributing to the experience of music, while Openness to experience and/or Extraversion may lead to more varied experience of emotions in response to music perceived as beautiful. Musicians may also be expected to report a wider range of impacts from music.’

**Comment #8**: The present study
p.9, l.203-205: It is difficult to understand what the research gap is. It can be easier for readers to understand if succinct summary is again here.

*Thank you for your careful review and comment. As requested, we have now made the gap very clear in previous sections throughout the manuscript. This means however that another summary here would be highly redundant.*

**Comment #9**: The present study
p.10, l. 221-223: ‘As for emotion and impact, text responses allow the reporting of rich and diverse responses that might not be possible if participants were provided only with pre-defined emotion labels and statements, e.g., [100,101].’
Specifically, this part should describe the limitations of the methods used in previous studies and how the methods used in this study can overcome those limitations. The same applies to text analysis. The weaknesses of text analysis and the advantages of the methods used in this study should be mentioned.

*Thank you for your comments. The limitations of the methods employed in previous studies have now been identified earlier when we describe them and here, we stress the advantages of the methods employed in this study, as follows;*

-Line 270, page 12.

Note: “[the advantage]”, ”[the limitation]” and highlighting have been added here for clarification only.

‘Specifically, regarding exploring the features of sound in music associated with beauty, text responses reveal listeners’ psychological impressions of both lower and higher- level features of sound [the advantage] in ways that might not be possible using automated feature extraction techniques [the limitation]. As for emotion and impact, text responses allow the reporting of rich and diverse responses [the advantage] that might not be possible if participants were provided only with pre-defined emotion labels and statements [the limitation], e.g., [100,101].’

**Comment #10**: Participants
p.11, l.242: What is ‘Prolific’?

*We’re sorry that we didn’t provide a clear description. Prolific is an online platform where researchers can recruit participants for their online studies. We have added this information to the manuscript;*

-Line 296, page 14.

‘A total of 81 participants..., having been recruited through the online participant recruitment platform, Prolific and...’

**Comment #11**: Survey
p.12, l.260-263:‘“Could you tell us the details of three pieces of music which you find to be very beautiful?” for the beautiful pieces block, and “Now, could you tell us the details of three pieces of music you listen to often but which you wouldn’t describe as beautiful?” for the not beautiful block.’
The phrase “listen to it often” is included when asking about songs that are not beautiful, but why is that? Given such frequencies of listening, wouldn't other factors such as familiarity are related to? Also, why didn't author(s) ask what “beautiful songs they listen to often” are?

*We included the phrase ‘listen to often’ for not beautiful pieces to prevent participants from conflating not beautiful music with disliked music. We wanted to make sure that participants provide not-beautiful pieces they choose to listen to as they choose to listen to beautiful pieces.*

*However, we agree that it may have helped reduce any effect of familiarity if we had requested that participants report beautiful music they listen to often to match our control condition. We now mention this as a limitation.*

-Line 1044, page 64.

‘Additionally, our study did not examine how familiarity or preference for the pieces listed by participants might have influenced their experience of musical beauty. Future studies could impose more control to ensure that participants’ level of familiarity and liking are maximally comparable between beautiful and not beautiful pieces.’

**Comment #12**: Analysis Text Responses to open-ended questions
p.15, l.319-325: Because coding greatly influences the results of this study, it is necessary to mention the reliability of coding. For example, indicators such as the kappa coefficient would be useful.

*Thank you for pointing this out. We have now included Cohen’s Kappa and prevalence-adjusted bias-adjusted Kappa (PABAK).*

-Line 386, page 18.

‘Cohen’s Kappa and PABAK values across codes (mean [range]) indicated moderate to very strong agreement: For Feature, Kappa = 0.78 [0.61-0.94], PABAK = 0.83 [0.55-0.97]; For Affect, Kappa = 0.85 [0.84-0.97], PABAK = 0. 87 [0.00-0.98]; For Impact, Kappa = 0.52 [0.13-1.00], PABAK = 0.90 [0.72-1.00].’

**Comment #13**: Results and Findings Pieces volunteered by participants.
p.17, l.358-374: It can be reasonable to assume that experiencing beauty has a significant influence on human behavior. However, if that is the case, why is it that many songs considered beautiful are classical music, which people do not listen to often? Conversely, why is rock music, which is considered less beautiful, listened to?

*Thanks for this interesting comment. We would argue, however, that even if beautiful music was listened to less frequently than music not found beautiful it would still have the ability to influence behaviour as much if not more. For instance, it is possible that beautiful music (which can often be classical music) is listened to more attentively than non beautiful music and as such has a more significant emotional impact. We, however, refrain from adding this speculation to the manuscript in the interest of brevity.*

**Comment #14**: Table 1. Examples of Responses from Feature Questions.
p.l8-20: Regarding Table 1, why were so many negative responses regarding music that is not beautiful obtained? The question asks about music that is “often listened to but not beautiful.” These responses appear to be impressions of music that the respondents do not like.
Why do respondents “often listen to” songs with such negative characteristics?
Do these responses indicate a gap between beauty and preference? Or did the participants list songs that are “generally considered beautiful”?

*Thank you for this question. We want to stress that negative feelings here include feelings like agitation and restlessness which while not positive, might be aesthetically valuable to a listener. In that vein, the occurrence of negative feelings may not be so surprising.*

*To clarify your last question, participants were asked to answer*

*1. sonic features that they think contribute to their experience of beauty in general*

*2-4. sonic features that they think contribute to their experience of beauty in the three pieces of music they listed*

*5. sonic features that they think contribute to their experience of a lack of beauty in general, and*

*6-8. sonic features that they think contribute to their experience of a lack of beauty in the three pieces of music they listed*

**Comment #15**: Discussion
p.37, l.652-660: The introduction did not clearly explain the research gap, making it difficult for readers to understand what new findings this study revealed. The introduction should clearly state the research gap and then summarize the main findings of this study in a concise manner at the beginning of the discussion in response to that gap.

*Thank you very much for your suggestion. We have now added the paragraphs summarising the research gaps and the findings of this study*;

-Line 815, page 53.

‘While the experience of musical beauty holds a prominent place in musical creativity, performance and listening e.g., [5–7], how it is experienced is insufficiently understood in terms of the contributing musical features (*Feature*), the range and patterning of feelings listeners report (*Affect*), and the impact the experience of musical beauty has on the listener (*Impact*). Also, relatively unclear is how all three vary depending on the listener’s musical training and personality traits.

Fig 8 summarises the findings across questions. With regard to musical features, our study extended previous work to reveal that while musical beauty is attributable to certain *Intrinsic features of the sound and music*, it is also related to *Cognitive structural factors* such as the degree of complexity, change and repetition, and to *Aesthetic criteria* the listeners apply including *Expressivity of the music* and *Performance qualities*. Further, with regard to emotion and impact, our study showed that musical beauty evokes *Calm and relaxing* feelings, *Sadness*, and the feeling of *Being moved and transported*, in addition to having high *Emotional resonance* with the listener and the ability to inspire. Finally, it was shown that musical beauty uniquely impacts listeners primarily by providing *Emotional help and bringing positivity.* An exploratory quantitative approach showed that musicianship and openness are positively associated with mentioning a greater array of contributing musical features, while musicianship and conscientiousness are related to more frequent mentions of musical beauty’s impact on *Musical influence*.

These findings significantly expand our understanding of the experience of musical beauty by highlighting previously unexplored aspects of musical beauty such as structural cognitive factors, epistemic emotions, long-term impact, and individual differences. In the following sections, we will discuss how our findings highlight the distinctive characteristics and occurrence of the experience of musical beauty. ’

**Comment #16**: Discussion
p.37, l.652-660: In the discussion, it is important to clearly state what new findings were revealed in this study that were not previously identified in the literature.
While the author(s) argue that the results of this study are consistent with many previous studies, they do not clearly state the novelty of this study. To help readers understand the novelty of this study, it is important to clearly explain what is new about this study.

*Thank you very much for pointing out this. To clarify the novelty of study, we added sentences or phrases in various places throughout Discussion, for example*;

-Line 868, page 56

‘To our knowledge, ours is the first study to qualitatively show that the well-established cognitive mechanisms underlie aesthetic experience to musical beauty.’

-Line 904, page 58

‘However, perhaps the most striking aspect of the affective profile participants associated with beautiful music, and one which has not been reported elsewhere, is ...’

**Comment #17**: Discussion
p.37, l.652-660: In the discussion, “beauty” and “preference” seem to be confused. Does the content that the author(s) are discussing as ‘beautiful’ music also apply to music that is “preferable but not beautiful”? Judging from the participants' responses, beauty seems to be associated with positive contents and, as a result, with preference. Therefore, analyzing these responses seems to be discussing beauty and the positive contents and preferences it contains as a single entity.
Indeed, it is possible to find something beautiful but not like it. This phenomenon appears to be similar to the distinction between “perceived emotion” and “felt emotion” often discussed in terms of musical emotions. Does beauty not have such a state? In any case, careful discussion is necessary about what the participants' responses actually represent.

*We are sorry to heard about this confusion. We’d never assumed that beautiful music is preferred nor not beautiful music is not preferred.*

*It is true that responses to beautiful pieces may be associated with positive contents, but this is also true to responses to not beautiful pieces as can be seen from Tables 1 to 3, suggesting that the experience of musical beauty is independent of listeners’ liking/preferences. Indeed, our participants’ responses revealed that participants do highly appreciate music even when they think it lacks beauty. In any case, we hope our carefully revised MS means this point is no longer a valid concern or source of confusion for the reviewer or future readers.*

**Comment #18**: Discussion
p.37, l.652-660: The analysis and results are wide-ranging and contain many contents related to beauty, making it difficult to grasp. It can be easier for readers to understand if the results of “beauty” obtained in this study could be modeled or diagrammed as a figure.

*Thank you for your suggestion. We agree and have now added a figure that highlights the key findings of our study (Fig 8 on page 54).*

**Fig 8. Summary of Study Findings**.

**Comment #19**: Discussion
p.37, l.662-663: The sub-heading is long and vague, so it should be made more concise and to the point.

*The sub-headings have been shortened as following;*

Beautiful music is evaluated in terms of its intrinsic, structural and aesthetic elements (Line 843, page 55)

Beautiful music elicits calm, sadness, being moved and support (Line 892, page 57)

Beautiful music impacts mood, musical motivation, but not identity (Line 952, page 60)

**Comment #20**: Discussion Beautiful music is characterised by its intrinsic features, cognitive structural factors and listeners’ application of aesthetic criteria
p.38, l.682-683: ‘while Complexity level is reminiscent of the inverted-U [92,93] and processing fluency theories [32].’
Please explain in detail. Have these theories been used to explain beauty in previous studies? Otherwise, is this a new finding of this study?

*This has been rephrased to clarify;*

-Line 863, page 56

‘The finding that the subjectively right level of complexity is required for beauty to be experienced aligns fully with the inverted-U theory [102, 121] and, to some extent, with processing fluency theories [38].’

*The theories mentioned in this section haven’t been applied to musical beauty, so we added the following sentence at the end of paragraph;*

-Line 868, page 56

‘To our knowledge, ours is the first study to qualitatively show that the well-established cognitive mechanisms underlie aesthetic experience in musical beauty.’

**Comment #21**: Discussion The affective profile of beautiful music: feeling calm, pleasurably sad, being moved and feeling emotionally supported
p.40, l.715-772: Because discussion about beauty and preferences is lack, it should be discussed.

*Thanks very much for your suggestion. Because we did not directly ask whether and to what degree the participants like each of six pieces of music they listed in the questionnaire, it is hard to discuss preference in the discussion in any substantive way. We have commented on this important point when discussing limitations of our study.*

-Line 1044, page 64

‘Additionally, our study did not examine how familiarity or preference for the pieces listed by participants might have influenced their experience of musical beauty. Future studies could impose more control to ensure that participants’ level of familiarity and liking are maximally comparable between beautiful and not beautiful pieces.’

**Comment #22**: Discussion The affective profile of beautiful music: feeling calm, pleasurably sad, being moved and feeling emotionally supported
p.42, l.760-761: ‘There are arguably two reasons why the pieces that listeners find beautiful might help them to deal with emotional difficulties.’
Do these conclusions apply to music that is “not beautiful but preferable”? Ultimately, as mentioned earlier, it is necessary to pay attention to what the participants' responses represent in order to draw conclusions.

*Since emotional help was a distinct feature of beautiful music (whether it is preferred or not), we would limit these claims to beautiful music. However we agree it is an interesting and important question.*

**Comment #23**: Discussion Beautiful music impacts mood, evokes the past, motivates musical
activities, but is not used to shape identity
p.43, l.782-784: ‘Another impact many mentioned is that listening to beautiful pieces was able to help them retrieve past events, people or feelings in memories, as discussed in the wide literature on music and autobiographical memories, e.g., [127].’
Isn't this a case of reverse causality? Could it be that we find certain songs beautiful because they are associated with specific memories? Or is it that beautiful songs are more likely to be associated with memories? In any case, evidence is needed to support this argument.

*We apologise that this sentence gave the impression that beautiful pieces tended to help listeners retrieve past memories more than not beautiful pieces. In fact beautiful and not beautiful music did so fairly equally and so to avoid confusion, the sentence has been rephrased as;*

-Line 965, page 61

‘There was a slight tendency for people to report that beautiful, compared to not beautiful, music encouraged engagement in further musical activities. However, few other impacts from music were specific to beautiful music. For example, many mentioned that listening to beautiful pieces was able to help them retrieve past events, people or feelings in memories, as captured in the code *Remembering the past*, but this was not any more the case than for not beautiful music: an interesting addition to the wide literature on music and autobiographical memories e.g., [134].’

**Comment #24**: Discussion Beautiful music impacts mood, evokes the past, motivates musical
activities, but is not used to shape identity
p.43, l.792-796: ‘Responses revealed that while listening to both beautiful and not beautiful pieces stimulate the listener’s musical curiosity and intelligence (such as the desire to understand the structure of the piece, enjoy its complexity, or discover novel musical expressions), listening to beautiful pieces tended to give rise to the desires to engage in further musical activities.’
This part seems to contradict the subsequent discussion. Ultimately, does beauty promote motivation, or is there some other mediating factor?

*Apologies for this seeming contradiction. We have rephrased the paragraph as shown below. Yes, beauty seems to promote motivation, but we cannot say from the findings of this study whether there are other mediating factors.*

-Line 971, page 61,

‘Similarly, listening to both beautiful and not beautiful pieces stimulated the listener’s musical curiosity and intelligence—the desire to understand the structure of the piece, enjoy its complexity, discover novel musical expressions, or to engage in further musical activities. This study extends earlier work that argues the desire to understand the piece and the desire to continue the experience often arise when beauty is experienced [15,47], by showing that such epistemic emotions can also be evoked by not beautiful pieces.’

**Comment #25**: Discussion Beautiful music impacts mood, evokes the past, motivates musical
activities, but is not used to shape identity
p.44, l.802-804: ‘Participants also reported that listening to not beautiful pieces influenced their musical preference for particular genres and helps shape their musical identity and identity in a broader sense, in line with previous studies [130–132].’
Why do not beautiful songs contribute to identity formation? How have previous studies discussed such tendency?

We can only speculate as to why beautiful music doesn’t contribute to identity formation. One reason, as we discuss in the manuscripts, is that experiences of beauty feel more universal than experiences of other music. They may thus contrast with music that listeners may, for instance, have discovered in their youth and may have used to build a sense of identify that they share with their peers. We believe the wide literature on music and identity formation support this idea and we now add more discussion points to this effect.

-Line 977, page 61

‘Finally, it is interesting to note that some impacts seemed to be greater for not beautiful than for not beautiful pieces. Specifically, participants reported that listening to not beautiful pieces influenced their musical preference for particular genres and helped shape their musical identity and identity in a broader sense (the code *Shaping musical taste and musical identity* in the theme *Musical influence*). Several studies have demonstrated music’s contribution to identity formation [135–137] and so a lack of forming either a musical or broader identity with music one finds beautiful is interesting, in seemingly resonating with the philosophical idea of the universality of beauty, e.g., [44]. This idea, supported by previous empirical data [15], states that people experiencing beauty tend to feel that others will also find the piece they consider beautiful to be beautiful. A potential inability of music experienced as beautiful being able to distinguish oneself from others may explain why this affordance is instead obtained from the other music people value and often choose to listen to.’

**Comment #26**: Discussion Individual differences in musical beauty experience
p.44, l.811-834: The reasons for the results differing from those of previous studies should be discussed in detail. Otherwise, it will be difficult for readers to understand what these results represent.

*Thank you for your suggestion. We have extended discussion of this and additional results in relation to findings from previous studies.*

-Line 991, page 62

‘Finally, our exploratory, quantitative approach revealed that both amateur musicians (Musicianship medium) and those who were high in Openness to experience mentioned a more extensive range of features contributing to musical beauty, compared to participants with lower or higher musical expertise, and lower in Openness to experience. This finding may be due to several factors: musicians’ enhanced perceptual and cognitive musical abilities [82–87] and their greater ability to explain music and musical experiences using technical terms [138]; and openness’s positive association with musical cognitive skills [96], along with a greater tendency to articulate a greater variety of perceptual processes [139] and to use more complex language [140]. ’

**Comment #27**: Discussion Individual differences in musical beauty experience
p.44, l.822-825: ‘For instance, professional musicians tended to mention Intrinsic features of sound and music (Feature) in beautiful music more frequently, reflecting musicians’ finer perceptual and cognitive musical abilities, e.g., [133], and their greater ability to explain music and their musical experiences using technical terms, e.g., [134].’
Does this tendency indicate that musicians and non-musicians have different perceptions of what constitutes “beauty”? If so, then the results obtained from the responses may simply reflect individual differences in the interpretation of the word “beauty,” rather than a specific ability to perceive music as beautiful.

*Thank you for this point. However, we have revised this section to accommodate updated findings (thanks to a suggestion from Reviewer 2) that indicate that both musicianship and openness explain participants’ mention of intrinsic features (Feature question), and that musicianship and conscientiousness are associated with reporting a wider range of musical influence from the experience of musical beauty (Impact question).*

**Comment #28**: Discussion
p.45, l.842-848: ‘Musical beauty seems to offer the listener not only an opportunity to appreciate music’s form, emotional expression and originality, but can also offer consolation and support in challenging times. Interestingly, while some intuitive trends were observed with regard to how individual differences influence the experience of musical beauty, our results were largely in keeping with Plato’s and Kant’s idea, supported by recent empirical work [11] that the experience of musical beauty (specifically features perceived as important, emotional responding, and psychological impact) is largely universal.’
Since the respondents' comments were so diverse, isn't it natural that there would be individual differences? Just because no individual differences were observed in terms of personality trait does not mean that it is an exaggeration to generalize beauty.

*Thanks very much for your comments. In light of additional findings, this section now reads as follows.*

-Line 1062, page 65

‘Interestingly, we also found that while there are no individual differences in terms of the variety of emotion characteristics listeners experience from musical beauty, musical expertise and some personality traits can influence the beauty experience; both in terms of the breadth of features of musical beauty recognised as important and the breadth of impact it has on people’s musical activities and musical being’

**Reviewer #2**: Thank you for the opportunity to review the manuscript entitled "Unpacking musical beauty: sound, emotion, and impact differences across expertise and personality" (Manuscript ID: PONE-D-25-24652), submitted to PLOS ONE.
This manuscript uses a mixed-methods approach to explore the concept of musical beauty, with a sample of 81 adult participants. Participants were asked to provide qualitative reflections on musical features, emotional responses and the long-term impact of pieces they considered beautiful, as opposed to pieces they liked but would not describe as beautiful. Through thematic analysis, the authors identified recurring themes associated with the perception of musical beauty. A subsequent quantitative analysis used Poisson regression to examine whether differences in musical training and personality traits predicted how often participants referred to specific themes when reflecting on beautiful versus non-beautiful music.

**General comments**:
Overall, I think the manuscript is well written, clearly organized, and engaging. I appreciated the way in which the authors combined qualitative insights with quantitative analysis, and really enjoyed reading the manuscript. In my opinion, the manuscript is almost ready for publication. I have provided some suggestions for minor revisions in the detailed comments below. which I hope the authors will find useful.

**Detailed comments**:

**Abstract**:
The abstract is clear and well-written. It is especially helpful that it immediately summarizes the key characteristics that according to the qualitative results distinguish music that is perceived as beautiful from music that is merely liked. To improve clarity, the authors could provide brief, concrete examples of the high-level features mentioned, such as specifying “a balanced level of complexity” rather than the more general “complexity level”.

*Thank you for this useful suggestion. However, having made some changes to the abstract to emphasise findings that are particularly novel to the study (on Reviewer 1’s recommendation), that sentence has been revised to read as follows*;

-Line 33, page 2

‘Thematic analysis of text responses showed that participants consider intrinsic features of the music, cognitive factors, and aesthetic criteria when judging the beauty of a piece.’

**Introduction**:
- The introduction is well-structured and provides a comprehensive overview of previous research into the concept of musical beauty. One area that could benefit from further clarification in my view is the distinction between beauty, aesthetic emotions (such as awe) and cognitive judgements about music. Since musical beauty is conceptualized differently in different studies (sometimes more as an aesthetic emotion and sometimes more as a cognitive judgement) these different perspectives could be introduced more explicitly at an earlier stage.

*Thank you for this important point. We agree with the need to explicitly define and differentiate between key concepts. Accordingly, we have revised the MS to include the sentences like:*

-Line 60, page 3

‘In addition to lacking a clear definition, also frequently debated is how beauty should be conceptualised. Beauty has been considered by some psychologists as a judgement towards an object [21,22], at the same time as it has been conceptualised as a particular, highly valued, feeling or emotion that an observer may experience when encountering an object [15,23]’

-Line 67, page 3

‘An aesthetic judgement is considered an output, along with emotion and preferences, of the processing of artworks and other objects. This form of processing is held to involve perceptual, cognitive, and affective components, e.g., [21,25,26],’

- Prior to discussing previous research on the characteristics of beautiful music, the authors could introduce the three main focus areas of the study (musical features, emotional responses and long-term impact) more clearly. Since other dimensions, such as cognitive responses (e.g. thoughts evoked by the music), could also be relevant, providing a brief rationale for why exactly these three areas were chosen would strengthen the framing of the study.

*Thank you for mentioning this. Reviewer 1 also asked us to more clearly state the research gap, so we have added a paragraph to make it clearer that these three things were addressing core research gaps we identified.*

-Line 88, page 4

‘However, while these recent studies [10,12] confirm that musical beauty is associated with certain sonic features and rich emotional content, there are still notable gaps in the literature. For instance, while Brattico and colleagues [10] compared sonic features of beautiful and not beautiful sections of three pieces of music, direct comparisons of *beautiful* with *not beautiful* pieces of music—as defined by the listener— are still rare. Moreover, although Fleckenstein et al’s study [12] tentatively indicates that musical beauty may have meaningful short-term impact on the listener—such as losing one’s sense of self, altered consciousness and being immersed—the long-term impact on individuals of experiencing musical beauty remains largely unexplored. Lastly, the extent to which the experience of musical beauty varies according to individual differences has not, to our knowledge, been examined, unlike in visual arts, where such questions have received significant attention [28,29].’

*We also agree that thought processes during beautiful music are relevant and of great interest. We therefore now recommend that this is pursued as a future research direction.*

-Line 1048, page 64

‘Finally, although the specific thoughts and metacognitive reflections associated with the experience of musical beauty are of considerable interest, they were beyond the scope of our study—and thus remain an area future research could fruitfully explore.’

- In line 61, beauty is described as a key factor in enabling positive aesthetic judgement. However, this may imply a directional relationship that is not clearly established, as the perception of beauty could also follow positive aesthetic judgement. Could you perhaps clarify this, or formulate both directions?

Thank you for this important point. We have clarified this as follows:

-Line 60, page 3

‘In addition to lacking a clear definition, also frequently debated is how beauty should be conceptualised. Beauty has been considered by some psychologists as a judgement towards an object [21,22], at the same time as it has been conceptualised as a particular, highly valued, feeling or emotion that an observer may experience when encountering an object [15,23]. Yet, other contemporary psychologists have conceptualised beauty as being one of a number of key criteria for arriving at a positive aesthetic judgement [3,9,24], where recognising beauty is generally associated with more positive aesthetic judgements. ’

- Line 86: It would be helpful to explain why only tension and energy are mentioned in the context of the Omigie et al. study. Were these the most commonly reported emotions in this study or the only emotions contributing to different emotional profiles of beautiful vs. not-beautiful music pieces?

*In this study by Omigie et al (2021), participants rated tension, energy, pleasantness, interest, and empathy experienced from listening to beautiful passages of music, but they did not give free text responses of emotions they experience from musical beauty. Tension and energy were the emotions that most distinctively distinguished different types of beautiful passages, which is why the manuscript mentioned those two.*

*All of that said, we have now revised the sentences describing that study, including removing mentions of associated emotions. This is to clarify to our reader that what makes that paper particularly valuable is showing how changes in musical elements may be important for the experience of beauty;*

-Line 120, page 6

‘Similarly, a study by Omigie et al [13] demonstrated that while beautiful passages or sections of music could can vary in characteristics, they are often characterised by change such as a drop in tempo and a reduction in polyphony in some cases, or increases in dynamics, pitch register, major tonality, and harmonic clarity, in others.’

- Line 96 – “Perceived beauty has been argued to be influenced by the ease with which a piece a piece can be cognitively processed” Here the direction of the effect could be clarified. Is beauty associated with greater or lesser processing ease?

*Thank you. We have now revised the text to clarify the direction of the effect*:

-Line 130, page 6

‘Indeed, according to the processing fluency theory [38], perceived beauty may be influenced by the ease with which a piece can be cognitively processed, where the easier it is to process the piece, the more beauty may be experienced.’

- When discussing the possible influence of musical expertise on the perception of beauty, it would be beneficial to introduce the concept of knowledge-based emotions at an earlier stage in the manuscript. Currently, this concept is only briefly mentioned in the discussion.

*Thank you for this suggestion. We have now introduced the concept of knowledge-based emotions as early as on line 160, page 8:*

‘Further, hinting at the additional role that epistemic emotions may play, Armstrong and Detweiler-Badell [47] argue that the desire to understand a piece and the satisfactory resolution of such desire through apprehending the piece, are essential for experiencing beauty’

Method:
- I recommend adding the age range of the participants to the sample description. If available, it would also be helpful to include more demographic information, such as the participants' country of residence and educational background. It would also be useful to state whether any eligibility criteria or filters were applied during the recruitment process. Since cultural background may influence perceptions of musical beauty, including these details would provide important context for interpreting the findings.

*Thank you for pointing out these important omissions. The age range, the participants' country of residence, and eligibility criteria have now been added. We unfortunately do not have information about participants’ educational background, and therefore could not add it.*

-Line 294, page 14

‘A total of 81 participants (female: *n* = 42, male: *n* = 36, non-binary/third gender: *n* = 2, Prefer not to say: *n* = 1; *M*_age_ = 36.30, *SD*_age_ = 15.73, range 18 to 72 years old) voluntarily took part in the survey, having been recruited through the online participant recruitment platform, Prolific and through social networks between 12 January and 13 March 2024. The only eligibility criterion for participation was to like listening to music. All participants were residents of the UK; however, 19.75% of the participants (n =16) were originally from other countries, including Italy: *n* = 4, Nigeria: *n* =4, the USA: *n* =2, Denmark: *n* =1, Hong Kong: *n* =1, Lithuania: *n* =1, New Zealand: *n* =1, Poland: *n* =1, and Sri Lanka: *n* =1.’

- Line 303 – “however, these measurements were not included in the current analysis.” Consider rephrasing this sentence to make it clear that only the EBS was excluded from the analysis (and not both EBS and TIPI)

*Thank you for your suggestions. It has now been rephrased as;*

-Line 361, page 17

‘Additionally, participants’ levels of engagement with artistic beauty, beauty in nature, and beauty in human morality were measured using an Engagement with Beauty Scale (EBS) by Diessner et al. [16], although the EBS data was not included in the current analysis.’

Analysis:
- The subheadings in the analysis section could be more specific. Consider using headings such as “Qualitative analysis of open-ended responses” and “Quantitative analysis of the influence of musicianship and personality traits”.

*We agree they could have been more informative. We have changed the subheadings mentioned as suggested. Since the other reviewer commented on the subheadings in Discussion section, we reviewed all subheadings in that section too.*

- Thematic analysis: The description of the coding process could be clearer. To me, it was not entirely clear whether only Author 2 initially coded all responses and then discussed the codes with others, or whether multiple authors independently coded the data. If multiple coders rated the same responses, it would be important to report an inter-rater reliability metric, such as Fleiss’ Kappa.

*We have now clarified the description of the coding process and have also reported both Cohen's kappa and Prevalence-Adjusted and Bias-Adjusted Kappa (PABAK) measures of inter-coder reliability. We used Cohen's kappa instead of the suggested Fleiss’ Kappa because the text responses were coded by two coders and calculated PABAK due to the high variability in code prevalence, with many codes being mentioned only rarely (e.g., the code ‘Originality’ (Feature question) was mentioned only 6 times across all participant responses, compared to ‘Emotional effect’, which was mentioned 119 times).*

-Line 374, page 17

‘We analysed text responses to questions related to the Feature, Affect, and Impact questions separately, using a codebook approach [113]. First, the researchers independently read the data several times to become familiar with it. Next, the first author coded 80 % of all responses, and created codes and a codebook that all authors then evaluated for appropriateness. Finally, the second author independently coded all responses following the codebook.

Cohen’s Kappa and prevalence-adjusted bias-adjusted Kappa (PABAK) was estimated per code (for the 80% of data coded by two authors) to assess inter-coder reliability and to identify any codes that might need refinement. PABAK was calculated along with Cohen’s Kappa since PABAK addresses the limitation that Cohen’s Kappa does not account for prevalence of codes [114]. PABAK was highly relevant in our dataset, where 8 out of 15 codes for the Feature question had an occurrence rate of less than 10 %, and 13 of 16 codes for both the Affect and Impact questions, had an occurrence rate of less than 10 % (See S1 Supporting Information). Cohen’s Kappa and PABAK values across codes (mean [range]) indicated moderate to very strong agreement: For Feature, Kappa = 0.78 [0.61-0.94], PABAK = 0.83 [0.55-0.97]; For Affect, Kappa = 0.85 [0.84-0.97], PABAK = 0. 87 [0.00-0.98]; For Impact, Kappa = 0.52 [0.13-1.00], PABAK = 0.90 [0.72-1.00]. ’

- Line 350 – “Poisson mixed effect models were run to examine the influence of music type and listener individual differences on these dependent variables of interest”: It took me some time to understand that the dependent variables were the themes found within the three core topics of features, emotions and impacts. This could be stated more clearly.

*Apologies that this section was not sufficiently clear. We have rephrased the section explaining the Poisson mixed effects models to clarify the dependent variables, and describe our updated modelling approach.*

-Line 402, page 19

‘To prepare the data for such modelling, we considered participants’ responses for each question separately. First, for each participant, for each text response, we determined whether a given code was relevant to that text response. We then collapsed across responses given by that participant (up to 4 possible responses given they could report on up to three pieces and/ or generally) and assigned the participant a count of one for that code regardless of how many times it was mentioned. Finally, to get our dependent variable (DV) we summed up the number of codes each participant had from each theme.

Our primary concern was on the level of breadth with which participants responded to each question, with an interest in any differences that may emerge on the theme level. Thus, two main Poisson mixed effects models were conducted on participants’ responses to each question. The first, simpler, model had number of codes as the DV and included musicianship level (high, medium, low) and scores on the five personality variables as fixed effects, with participant ID included as a random effect. The second, more complex model was the same but additionally included theme as a fixed effect that could interact with each of the (individual difference) fixed effects in the first model. Poisson mixed effects models were chosen due to the data being count data. All analyses were performed in R [115], using the *glmer* function from the lme4 package [116].’

- Line 351-356 “with participant ID and pieces included as random effects”: From the phrasing of the open-ended questions, it appears that participants were asked within the same question to describe features (in the other questions emotions or impacts) across three selected musical pieces, as well as more generally for music they consider beautiful or not beautiful. If so, it may have been difficult to distinguish whether the responses referred to the specific pieces or the category as a whole. This raises the question of whether it would have been more appropriate to count themes at the condition level (beautiful vs. non-beautiful) rather than for each individual piece. Aggregating theme counts by condition could also reduce model complexity of the Poisson regressions. However, I may have misunderstood this part of the procedure. If so, a clearer explanation in the manuscript would be helpful.

*Thank you very much for your comment. As you recognised, participants were able to give up to four text responses for each condition (beautiful or not beautiful) as a result of being invited to talk about pieces specifically and/ or in general. While there was a text box for each piece and for the general comments (allowing us to be able to distinguish these ) we now see collapsing across pieces and the general comment may be more parsimonious (given the freedom we allowed participants when making their responses)*

*The updated approach can be seen in response to the previous comment.*

Results:
- Figure 1/Table 1: To me, it was not immediately obvious that the quotes in Table 1 are not organized by the individual codes listed in Figure 1. This could be made clearer. One way to achieve this would be to present one representative quote per code, or alternatively, to label each quote with the corresponding code name.

*Thank you for your suggestions. All quote IDs were re-labelled with the corresponding code name. e.g., “F-Building Blocks: Timbre-B1". We also examined the data and qualitative analysis, and divided the code “Building blocks” into six separate codes to more finely illustrate the different aspects of musical features that participants mentioned.*

- Table 5 & 6: Given the relatively small sample size, using Poisson regression models with 15 predictors could be too ambitious and risk overfitting. There may be alternative modelling strategies worth exploring. For instance, computing difference scores (i.e., the frequency of the theme being mentioned in the beautiful music condition minus the frequency in non-beautiful music condition) could streamline the analysis by eliminating the need for interaction terms. But this approach would likely require a linear regression model instead of a Poisson model, depending on the distribution of the difference scores. Another option would be to analyze theme frequencies only for the beautiful music condition, reducing model complexity and possibly yielding clearer insights. Although I am not a specialist in mixed Poisson regression, I would like to offer these suggestions as possible alternatives that might help to address the models' complexity relative to the sample size.

*Thank you again for your comments regarding the analysis approach. We see now the models carried out were potentially too numerous and too complex in the first instance. We have therefore sought to tackle both of these potential issues. First, we have now reduced the number of models to be two per question (one main analysis, and a further exploratory analysis), and have also reduced the data examined to focus on responses to beautiful music.*

*Specifically, in a simple model, we now take all mentions of codes nested within each theme, into one count and use only the musicianship and 5 personality traits scores as fixed effects, with participants’ ID as a random effect. A second model then allows Theme to interact with each of these individual difference fixed effects to allow exploration of how individual differences effects may be influenced by the themes in question.*

*Here, we note that complexity reduced by not adding beauty type (beautiful, not beautiful) as an interacting variable is somewhat re-introduced by making theme a fixed effect that interacts with the individual difference variables. However, we believe this model complexity is less of a concern, given the more exploratory nature of the secondary models.*

*This is explained in the paragraphs here*:

-Line 393, page 18

‘Specifically, for each question, we modelled how musicianship level and personality scores predicted the number of codes present in participants’ responses as they described their experience of musical beauty. The assumption was that the number of codes reflect the breadth of aspects involved in each participant’s experience. To give an example, musicians might be expected to report a wider breadth (and therefore have higher count) of musical features and impacts, just as participants high in Openness to experience and Extraversion might be expected to report wider-ranging (and therefore have a higher count of) affective responses.

To prepare the data for such modelling, we considered participants’ responses for each question separately. First, for each participant, for each text response, we determined whether a given code was relevant to that text response. We then collapsed across responses given by that participant (up to 4 possible responses given they could report on up to three pieces and/ or generally) and assigned the participant a count of one for that code regardless of how many times it was mentioned. Finally, to get our dependent variable (DV) we summed up the number of codes each participant had from each theme.

Our primary concern was on the level of breadth with which participants responded to each question, with an interest in any differences that may emerge on the theme level. Thus, two main Poisson mixed effects models were conducted on participants’ responses to each question. The first, simpler, model had number of codes as the DV and included musicianship level (high, medium, low) and scores on the five personality variables as fixed effects, with participant ID included as a random effect. The second, more complex model was the same but additionally included theme as a fixed effect that could interact with each of the (individual difference) fixed effects in the first model. Poisson mixed effects models were chosen due to the data being count data. All analyses were performed in R [115], using the *glmer* function from the lme4 package [116].’

**Discussion**:
- One area that could be expanded upon is the comparison of the present results with those of Fleckenstein et al. (2025). While the authors reference this study when discussing specific codes or themes, a more direct comparison of the relative prominence of themes identified in both studies would be valuable.

*Thank you. We have now carefully revisited the discussion to add more comparisons of the present results with those of Fleckenstein et al. (2025) where applicable. However, due to the differences in study designs between Fleckenstein et al. (2025) and our studies, and the fact that Fleckenstein et al. (2025) did not extract themes or elaborate on their findings, it is not straightforward to make a direct comparison at a macro-level (e.g., which themes or codes are similar, different, or missing in one study). We have nevertheless referred to this study as much as possible when discussing the results as we agree it is a highly relevant one to compare our study to*

*Added citation of Fleckenstein et al. (2025):*

-Line 917, page 58

‘physical reactions (e.g., chills and goosebumps) and the feeling of being transported have previously been associated with musical beauty [12].’

- Line 752-759: The link between musical beauty and emotion regulation is very interesting. Perhaps the authors could elaborate more on this point. For instance, different types of music (beautiful versus not beautiful) might serve distinct functions in mood regulation. High-energy, less beautiful music might be more suitable for emotional release or catharsis (e.g. discharging tension or negative emotions), whereas low-energy, beautiful music may be better suited to emotional reflection or finding emotional comfort.

*Thank you for your encouragement to elaborate on this finding. We have now discussed these potentially distinguishable links between musical beauty and emotion regulation:*

-Line 928, page 60

‘In line with the well-documented finding regarding the usage of music as an effective tool to manage and regulate one’s own mood and emotions, e.g., [62,130,132,133], many participants noted that listening to both beautiful and not beautiful pieces tended to change their affective state for the better, converting their negative mood or thoughts into positive ones (the code *Changing mood and feelings* in the theme *Psychological impact*). This was, however, more frequently mentioned in relation to beautiful pieces, which may indicate that experiencing beauty creates conditions for the listener’s mood to be more easily changed. Here it is interesting to consider potential differences in how beautiful and not beautiful pieces change the listener’s mood for the better. For beautiful pieces, this could be due to their soft and quiet sound, slow tempo and the resultant calmness, relaxation and consolation they bring, while for not beautiful pieces, this could be due to their louder sound, faster tempo and energising features, which may provide an emotional outlet for the listener.’
