# Supplementary material for: Unpacking musical beauty: Sound, emotion, and impact differences across expertise and personality
Source: PLoS One. 2025 Nov 14;20(11):e0335905. doi: 10.1371/journal.pone.0335905 (PMC12617921; doi:10.1371/journal.pone.0335905)
Supplement: Attachment — Submitted filename: Response_Letter2.docx [file pone.0335905.s005.docx]

Response_Letter2

Dear reviewers,

Thank you very much for the time taken to read our manuscript again. We appreciate the reviewers’ suggestion, and the manuscript has been revised accordingly, with modifications that enhance readability. Our detailed responses to each comment are provided below.

Reviewer #1:
**Comment #11: Survey**
p.12, l.260-263:‘“Could you tell us the details of three pieces of music which you find to be very beautiful?” for the beautiful pieces block, and “Now, could you tell us the details of three pieces of music you listen to often but which you wouldn’t describe as beautiful?” for the not beautiful block.’
The phrase “listen to it often” is included when asking about songs that are not beautiful, but why is that? Given such frequencies of listening, wouldn't other factors such as familiarity are related to? Also, why didn't author(s) ask what “beautiful songs they listen to often” are?

Response:
We included the phrase ‘listen to often’ for not beautiful pieces to prevent participants from conflating not beautiful music with disliked music. We wanted to make sure that participants provide not-beautiful pieces they choose to listen to as they choose to listen to beautiful pieces.
However, we agree that it may have helped reduce any effect of familiarity if we had requested that participants report beautiful music they listen to often to match our control condition. We now mention this as a limitation.
-Line 1044, page 64.
‘Additionally, our study did not examine how familiarity or preference for the pieces listed by participants might have influenced their experience of musical beauty. Future studies could impose more control to ensure that participants’ level of familiarity and liking are maximally comparable between beautiful and not beautiful pieces.’

Comment #11: Round2
The implications of this instruction should be considered in interpreting the results. The author(s) mentioned the association between beauty and processing fluency. Since familiarity correlates with processing fluency, the instruction “listen to it often” itself may be a factor related to beauty. If control for factors were to be exercised, why wasn't the instruction phrased as “beautiful music you listen to often”? Although beauty and liking are not entirely independent, the results derived from this instruction warrant more careful discussion. Among the two factors—beautiful/not beautiful, listen often/don't listen often—wouldn't “not beautiful and not listened to it often” be considered the least beautiful music?

***We would not want to argue that "Not beautiful and not listened to it often” music is the least beautiful because that would be relying heavily on the processing fluency account of beauty, when in fact we are keen to hear from participants themselves what music they find beautiful and not beautiful. However, we agree that in accordance with the processing fluency theory, the more music is listened to, the more it may be experienced as beautiful and less listened to the more it may be considered not beautiful. We also now see that, therefore, when we specify non beautiful pieces be pieces they listen to often, we may be leading them to mention pieces that while not beautiful, may still have decent levels of processing fluency for the listener.

As previously mentioned, we wanted to ensure that our control conditions included pieces of music that people wanted to and are likely to be hearing in everyday life. We, in other words, wanted to provide a naturalistic comparator group for our beautiful music condition i.e., we did not want to compare our beautiful music to the ‘ugliest’ music people can think of which they and other may not listen to in everyday life. We realise that using the word listen to often in not beautiful condition may introduce potential differences in the frequencies of music in the two conditions, but think that the benefits of our approach may outweigh negative in this case. We add this rational and justification even more clearly in our manuscript whilst leaving in the sections where we describe our limitations.

Lines 321-324, p. 15: Procedure

“We specified “listen to often’ when referring to not beautiful music to ensure that participants did not conflate ‘not beautiful’ with ‘disliked’ or ‘ugly’ music they would normally try to avoid, but rather considered not beautiful music that they nevertheless engage with in everyday life.”

Lines 1027-29, p.63: Limitations

“the use of the phrase “listen to often” only in not beautiful block in the questionnaire may have introduced differences in familiarity between beautiful and not beautiful music.”


**Comment #13: Results and Findings Pieces volunteered by participants.**
p.17, l.358-374: It can be reasonable to assume that experiencing beauty has a significant influence on human behavior. However, if that is the case, why is it that many songs considered beautiful are classical music, which people do not listen to often? Conversely, why is rock music, which is considered less beautiful, listened to?

Response:
Thanks for this interesting comment. We would argue, however, that even if beautiful music was listened to less frequently than music not found beautiful, it would still have the ability to influence behaviour as much if not more. For instance, it is possible that beautiful music (which can often be classical music) is listened to more attentively than non beautiful music and as such has a more significant emotional impact. We, however, refrain from adding this speculation to the manuscript in the interest of brevity.

Comment #13: Round2
This explanation requires further elaboration. One motivation for playing an instrument is a strong emotional experience, and certainly, the beauty of music is involved in such experiences. However, what is crucial here is the emotional experience itself—is beauty a necessary condition? Ultimately, as with the previous comment, beauty and personal preference are intertwined, so it is necessary to clarify these concepts before proceeding with the discussion.

***The section that the reviewer is referring to describe the frequencies with which beautiful and not beautiful music come from different genres. We think the reviewer, however, is keen to understand why music like rock, even if it’s not beautiful, may lead people to want to play music (that is why not beautiful music may impact behaviour). We think this is an interesting question, and to be clear we think, especially based on our results, that music does not have to be beautiful to impact behaviour. In other words, beauty is not a necessarily condition to impact behaviour and that preferred music can have this impact too. We point the reader to where this is clear from our manuscript.

For example,

Lines 683-695, p.45

“**Memory and the Self**

Participants’ comments demonstrated that while both beautiful and not beautiful music helped them connect with their lives, not beautiful music showed a higher tendency for codes related to everyday use and identity (57.14% to 100%). Participants described how both beautiful and not beautiful lead them to relive memories (I-“Remembering the Past”-B11, I-“Remembering the Past”-NB11), of past situations in which they listened to and/or performed the piece (I-“Remembering the Past”-B11), and with associated memories that were not always positive (I-“Remembering the Past”-B12, I-“Remembering the Past”-NB12). However, not beautiful music especially was described as serving as a practical aid to daily life activities, from housework, to the daily commute, and even to sleep (I-“Practical Chore”-B13, I-“Practical Chore”-NB13). Similarly, not beautiful music exclusively was described as having shaped the participants’ identity or personality (I-“Life Soundtrack”-NB14, Beautiful = 0%, Not Beautiful = 100%).”

Lines 704 – 712, p. 48

“Finally, it is important to note that participants reported not beautiful music especially (Beautiful = 35.71%, Not Beautiful = 64.29%) as having stimulated their musical curiosity (I-“Stimulating Curiosity”-NB18) and having widened their repertoire or interest (I-“Stimulating Curiosity”-B18). It is also notable that (similar to beautiful music never being reported as having shaped personal identity (see *Memory and the self*)), there was a greater tendency for not beautiful, compared to beautiful (Beautiful = 30.00%, Not Beautiful = 70.00%), to be reported as having shaped the participants’ musical taste (I-“Musical Taste and Identity”-B19, I-“Musical Taste and Identity”-NB19) and identity (I-“Musical Taste and Identity”-B20, I-“Musical Taste and Identity”-NB20). ”

**Comment #14: Table 1. Examples of Responses from Feature Questions.p.l8-20**: Regarding Table 1, why were so many negative responses regarding music that is not beautiful obtained? The question asks about music that is “often listened to but not beautiful.” These responses appear to be impressions of music that the respondents do not like.
Why do respondents “often listen to” songs with such negative characteristics?
Do these responses indicate a gap between beauty and preference? Or did the participants list songs that are “generally considered beautiful”?

Response:
Thank you for this question. We want to stress that negative feelings here include feelings like agitation and restlessness which while not positive, might be aesthetically valuable to a listener. In that vein, the occurrence of negative feelings may not be so surprising.

Comment #14: Round 2
Please incorporate the content here into the body text.

***Thank you. We realise now that it is important to mention this as you suggest. It has been added in the main text;

Lines 872-877, p. 56

“Negative emotions (such as restless, agitation, angst, hauntedness, frustration) were also reported, primarily in response to not beautiful pieces. The fact that participants chose to listen to music that evoked such emotions suggests that these pieces may hold aesthetic value despite not being considered beautiful and that experiencing these negative emotions through music may function as an emotional outlet, as reported in previous studies [126, 127].”

***

Reviewer #2: Dear authors,
Thank you for your additional work on the manuscript entitled “Unpacking musical beauty: sound, emotion, and impact differences across expertise and personality" (Manuscript ID: PONE-D-25-24652). Overall, I believe the manuscript has improved considerably, and I do not have any major concerns at this stage.

However, I have a few minor suggestions that may help strengthen the final version.
**1. Language use & style**: The manuscript is quite lengthy, and some sections are repetitive. For example, the sentence “In addition to lacking a clear definition, also frequently debated is how beauty should be conceptualised.” repeats the same idea. Similarly, in this passage the word "recognized" is repeated in consecutive sentences: "Here, it is recognised that it may be a positive experience for the listener when a piece of music unfolds as they anticipated [40,41]. However, it is also increasingly recognised that a degree of unexpectedness, surprise, or novelty in music can lead to greater feelings of pleasure.” These are just a couple of examples, but there are other similar instances throughout the text. I recommend carefully reading through the text with an eye toward tightening the language and reducing redundancy. You might also consider having a native English speaker review the manuscript to improve clarity and flow.

***Thank you for this comment. We have now carefully read and edited the manuscript. We hope the revised version has improved readability.

**2. Long-term influences**: The introduction mentions the long-term impact of experiencing beauty in music as a novel feature of this study. However, this aspect is not revisited in depth later in the manuscript. It is also unclear from the questionnaire whether participants were prompted to reflect on short-term or long-term effects. To clarify, it would be helpful to state in the introduction that the study explores both immediate and potentially lasting impacts. Consider referencing this again in the results and/or discussion.

*** Thank you for this. The questionnaire did not specify whether participants should reflect on short- or long-term impact. It was during the coding process we realised that it may be useful to separate these out. We agree with that the reviewer’s suggestion to be more consistent across the three sections of the manuscript. Therefore, we now, as advised, mention in the introduction that the impacts participants' report may be expected to range from shorter- or longer-term impacts and also, in the discussion, we emphasise that one might want to consider the impact of beauty in this nuanced way.

Lines 192-194, p.9: The Impact of experiencing beauty in music

“However, while it seems plausible that these shorter- and longer-term impacts may emerge from experiences of musical beauty specifically, this has not been examined in the extant literature on musical beauty.”

Lines 253-256, p. 11: The present study

“..., this study explored how the experience of musical beauty can be understood in terms of..., and the impact of musical beauty, including its potential short- and long-term effects.”

Lines 338-341, p. 17: Procedure

“Although this study aims to explore both short- and long-term impacts of experiencing musical beauty, participants were not instructed to report impacts as short- or long-term since it is difficult to specify these systematically.”

Lines 932-934, p.59: Discussion

“In line with the well-documented finding ..., many participants reported what could be considered short-term impacts, noting that listening to both beautiful and not beautiful pieces...”

Lines 965-967, p. 60

“The potential difficulty in distinguishing oneself from others through beautiful music may explain why the affordance of a longer-term identity is instead obtained from the other music people value and often choose to listen to.”

**3. Discussion**: I felt that the new discussion section headings were too long and complex. To improve readability and structure, I suggest simplifying them. For example: “Intrinsic, structural, and aesthetic elements of beautiful music”, “Emotional responses to beautiful music”, “Short- and long-term impacts of beautiful music”, “Individual differences in experiences of beauty”

*** Thank you for your suggestion. We have simplified the headings as following.

- Beautiful music is evaluated in terms of its intrinsic, structural and aesthetic elements

-> **The intrinsic, structural, and aesthetic determinants of beautiful music**

- Beautiful music elicits calm, sadness, being moved and support

-> **The characteristic emotional profile of beautiful music**

- Beautiful music impacts mood, musical motivation, but mot identity

-> **The scope and the nature of beautiful music impacts**

- Individual differences in musical beauty experience

-> **Individual differences in experiences of musical beauty**

**4. Figure 8 & Abstract**: For Figure 8, consider providing a more balanced level of detail across all three sections. In the section "What beautiful music makes people feel" you list specific emotional responses reported by participants. However, in "What makes music beautiful", you only list broader categories (e.g., timbre, melody) without providing concrete examples of what was perceived as beautiful. Similarly, the abstract mentions emotional and impact-related findings but does not touch on the specific musical features that participants associated with beauty. Since many readers may only read the abstract or jump directly to the discussion and this nice figure, clearly and concisely summarizing all key findings in both Figure 8 and the abstract would strengthen the accessibility and impact of your work.

*** Thank you for your helpful suggestion. We have added the more details of musical features in both Figure 8 and the abstract.

Fig 8. Page 53

Lines 32-36, p. 2: Abstract

“Thematic analysis of text responses showed that participants consider intrinsic features of the music (mellow timbre, pleasing melodies and harmonies, slow tempo), cognitive factors (degree of change, complexity levels, and repetition), and aesthetic criteria (emotional effects, presence of a message, expressivity) when judging the beauty of a piece.”

**5. Limitations**: Another potential limitation you might mention is the within-subjects design. Asking participants to reflect on beautiful and non-beautiful music may have encouraged them to emphasize the differences between the two, which could have influenced their responses. A future between-subjects design could have yielded different results and potentially reduced this contrast effect.

*** Thank you for this important point. It has been added to the manuscript as follow;

Lines 1024-27, p. 63: Limitations

“Similarly, the within-subjects design we employed, in which the same participants answered the questions regarding both beautiful and not beautiful music, may have artificially sharpened contrasts between responses—a between-subjects design could mitigate this.”
